# PAX-D: study protocol for a randomised placebo-controlled trial evaluating the efficacy and mechanism of pramipexole as add-on treatment for people with treatment resistant depression

**DOI:** 10.1136/ebmental-2021-300282

**Published:** 2021-11-22

**Authors:** Sheena Kristine Au-Yeung, James Griffiths, Sophie Roberts, Chloe Edwards, Ly-Mee Yu, Rafal Bogacz, Jennifer Rendell, Mary-Jane Attenburrow, Stuart Watson, Fiona Chan, Andrea Cipriani, Anthony Cleare, Catherine J Harmer, David Kessler, Jonathan Evans, Glyn Lewis, Ilina Singh, Judit Simon, Paul J Harrison, Phil Cowen, Milensu Shanyinde, John Geddes, Michael Browning

**Affiliations:** 1 Department of Psychiatry, University of Oxford, Oxford, UK; 2 Department of Primary Care Health Sciences, University of Oxford Nuffield, Oxford, UK; 3 Brain Network Dynamics Unit, University of Oxford, Oxford, UK; 4 Oxford Health NHS Foundation Trust, Oxford, UK; 5 Translational and Clinical Research Institute, Newcastle University, Newcastle upon Tyne, UK; 6 Inpatient Services, Cumbria, Northumberland, Tyne and Wear NHS Mental Health Trust, Northumberland, UK; 7 Independent Researcher, Unknown, UK; 8 Centre for Affective Disorders, Department of Psychological Medicine, Institute of Psychiatry, Psychology and Neuroscience, King’s College London, Bristol, UK; 9 Bristol Medical School, Bristol Population Health Science Institute, University of Bristol, Bristol, UK; 10 Division of Psychiatry, Faculty of Brain Sciences, University College London, London, UK; 11 Department of Health Economics, Medical University of Vienna, Wien, Austria

**Keywords:** depression & mood disorders, adult psychiatry, psychiatry

## Abstract

**Introduction:**

Clinical depression is usually treated in primary care with psychological therapies and antidepressant medication. However, when patients do not respond to at least two or more antidepressants within a depressive episode, they are considered to have treatment resistant depression (TRD). Previous small randomised controlled trials suggested that pramipexole, a dopamine D2/3 receptor agonist, may be effective for treating patients with unipolar and bipolar depression as it is known to influence motivational drive and reward processing. PAX-D will compare the effects of pramipexole vs placebo when added to current antidepressant medication for people with TRD. Additionally, PAX-D will investigate the mechanistic effect of pramipexole on reward sensitivity using a probabilistic decision-making task.

**Methods and analysis:**

PAX-D will assess effectiveness in the short- term (during the first 12 weeks) and in the longer-term (48 weeks) in patients with TRD from the UK. The primary outcome will be change in self-reported depressive symptoms from baseline to week 12 post-randomisation measured using the Quick Inventory of Depressive Symptomatology Self-Report (QIDS-SR16). Performance on the decision-making task will be measured at week 0, week 2 and week 12. Secondary outcomes include anhedonia, anxiety and health economic measures including quality of life, capability, well-being and costs. PAX-D will also assess the adverse effects of pramipexole including impulse control difficulties.

**Discussion:**

Pramipexole is a promising augmentation agent for TRD and may be a useful addition to existing treatment regimes. PAX-D will assess its effectiveness and test for a potential mechanism of action in patients with TRD.

**Trial registration number:**

ISRCTN84666271

## Introduction

When patients do not respond to at least two or more antidepressants within a depressive episode, they are considered to have treatment resistant depression (TRD). This accounts for roughly 20%–30% of all depressed patients and is a leading cause of morbidity and workdays lost. Currently there is a shortage of effective pharmacological options for patients who fall within this category.^1^

The large, pragmatic Sequenced Treatment Alternatives to Relieve Depression (STAR*D) trial suggested that the chances of remission with conventional pharmacological approaches for patients with TRD are less than 15%.^2^ A systematic review^3^ indicated that the best, current, evidence-based treatment is addition of atypical antipsychotic medications such as aripiprazole or quetiapine. However, these agents are only moderately effective and have high dropout rates associated with adverse effects. The extent and frequency of these adverse effects (sedation, weight gain and movement disorders) mean that this ‘atypical augmentation’ is widely disliked by patients. Another option is adding lithium to an antidepressant, but the evidence base is limited and lithium is poorly tolerated, potentially toxic and again disliked by patients. No new antidepressant is more effective than tricyclic antidepressants which were discovered serendipitously over 60 years ago. The pharmaceutical industry has largely withdrawn from this area because of the difficulty of making real advances.

There is preliminary evidence that the dopamine agonist, pramipexole, could represent an important advance in TRD. A systematic review by Tundo *et al* 4 identified five completed randomised controlled trials (RCTs), three open-label trials and five observational studies of pramipexole for patients with major depressive episodes and reported beneficial effects of pramipexole for patients with unipolar and bipolar depression. An RCT by Cusin *et al* 5 with 65 unipolar TRD patients found significant benefit of pramipexole, administered at a relatively low average dose of 1.3 mg/day, over placebo on a continuous outcome symptom measure but not in terms of response (40% vs 27%). Another RCT by Corrigan *et al* 6 examining pramipexole monotherapy in non-treatment resistant unipolar depression found that 1 mg was superior to placebo, while 0.375 mg was ineffective and 5 mg caused very high drop out. A very small study by Franco-Chaves *et al* 7 (n=13 per group) of unipolar patients resistant to a single previous antidepressant randomised to receive pramipexole monotherapy (target dose=2.25 mg), pramipexole combined with escitalopram or escitalopram monotherapy, found no statistical difference between the study groups, although only four patients in the combined group completed the study. Two RCTs of bipolar depression by Zarate *et al* 8 and Goldberg *et al* 9 found that patients on pramipexole (average dose of 1.7 mg) were more likely to have better therapeutic response than those on placebo. A case series of TRD patients treated in the US by Fawcett *et al* 10 reported very good therapeutic responses to pramipexole augmentation of antidepressant therapy in 42 patients with depression at a mean dose of around 2.5 mg/day. However, given the small sample sizes and inconsistent results summarised above, the review by Tundo *et al* 4 concluded that adequately powered RCTs of pramipexole for depression are still needed.

The target dose of pramipexole used in PAX-D will be 2.5 mg/day as a single dose. The target dose was selected to be above the average achieved dose of the Cusin *et al* study^5^ and between the effective 1 mg/day dose and non-tolerated 5 mg/day dose of the Corrigan *et al* study,^6^ while keeping within the smPC upper limit of 4.5 mg/day. The dosing schedule, with medication administered once daily rather than in divided doses as in Cusin *et al*,^5^ followed that reported for participants under 45 years of age in the Fawcett *et al* case series.^10^

Pramipexole is of particular interest because it is a selective dopamine D2/D3 receptor agonist and therefore pharmacologically distinct from currently available antidepressants which mostly act to increase levels of serotonin in the synapse. Experiences of low motivation and anhedonia are thought to be related to the function of the central dopaminergic system which is not specifically targeted by current medicines.^11^ Dopaminergic signalling is believed to be required when learning about rewarding outcomes.^12^ Previous studies of pramipexole in patients with Parkinson’s disease^13^ and bipolar disorder^14^ completing reward learning tasks indicated that it acted to increase reward sensitivity (ie, to cause patients to treat rewarding outcomes as if they were more valuable). This raises the possibility that increased reward sensitivity is a cognitive mechanism by which pramipexole improves symptoms of depression. Pramipexole is also studied in an ongoing trial about TRD in bipolar disorder.^15^

### Objectives

The primary objective of PAX-D is to assess the efficacy of adding pramipexole to conventional antidepressant medication for treating symptoms of depression. Pramipexole will be administered under double-blind, placebo- controlled conditions in patients with TRD. Second, PAX-D will examine the effect of pramipexole on reward sensitivity and whether this may predict treatment outcome. A decision-making task^16^ will be used in the current trial to measure the impact of pramipexole on reward sensitivity. The relationship between pramipexole treatment, change in reward sensitivity and symptomatic response will be formally assessed using a mediation analysis. Further, the trial will estimate the degree to which baseline and initial changes in reward sensitivity are able to predict response to pramipexole, providing a first test of the potential for these measures to be deployed in the selection of treatments for TRD patients. Finally, PAX-D will conduct an economic analysis of pramipexole and its longer-term effect on quality of life, capability, well-being, functioning and costs. Finally, the acceptability and tolerability of pramipexole will be assessed across the course of treatment. All objectives are summarised in table 1.

**Table 1:** Study objectives (from https://www.isrctn.com/ISRCTN84666271)

Objectives	Outcome measures	Time points
Primary objective	Primary outcome	
To compare the efficacy of pramipexole and placebo at 12 weeks postrandomisation	Improvement (change from baseline) of depressive symptoms measured on the QIDS-SR_16_	Week 1–12
Secondary objectives	Secondary outcomes	
To compare the tolerability and safety of pramipexole and placebo during the 48-week treatment phase	Tolerability assessed by: Termination of trial treatment due to intolerance Adverse reactions TSQM-9 Safety–emergence of new symptoms: ALTMAN (manic symptoms) QUIP-RS (impulse control) Suicidal ideation (QIDS-SR_16_)	Weeks 1–48
To compare the effect of pramipexole and placebo on reward sensitivity	Change in reward sensitivity parameter from model fitted to learning/decision making task between baseline, week 2 and week 12	Baseline, week 2, week 12
To test the degree to which change in reward sensitivity mediates the 12 weeks response to pramipexole of both depressive, and specifically anhedonic, symptoms	Change in QIDS-SR_16_ and SHAPS scores between baseline and week 12 and change in reward sensitivity between baseline and week 2	Baseline, week 2, week 12
To compare the extent to which an increase in reward sensitivity predicts therapeutic response	Change scores in the learning/decision making task at 2 weeks and the change in the QID-SR_16_ at 12 weeks	Week 2, week 12
To explore the extent to which reward sensitivity at baseline predicts therapeutic response	Baseline scores on the learning/decision making task and the change in QIDS-SR_16_ at 12 weeks	Baseline, week 12
To explore the extent to which level of anhedonia at baseline predicts therapeutic response	Baseline scores on SHAPS and change in the QIDS-SR_16_ at 12 weeks	Baseline, week 12
To compare the effect of pramipexole and placebo on the trajectory of symptoms of depression	QIDS-SR_16_ scores collected weekly across 48 weeks of the trial	Weekly for week 1–48
To compare the effect of pramipexole and placebo on response and remission rates, using the QIDS-SR_16_ at twelve weeks	QIDS-SR_16_ response, defined as a reduction of <50% of baseline scores at week 12, remission as a score of <5 at week 12	Baseline, week 12
To compare the impact of pramipexole and placebo on symptoms of anhedonia, anxiety and clinician rated depression	Change scores for the SHAPS, GAD-7 and QIDS-C between baseline and week 12	Baseline, week 12
To compare the impact of pramipexole and placebo on functional outcome over the 48 weeks of treatment	Change scores for the WSAS-screener between baseline and week 48	Baseline, weeks 12, 24, 36 and 48
To determine the impact on quality of life and capability well-being of pramipexole relative to placebo over 48 weeks	Change in the following over 48 weeks: EQ-5D-5L ICECAP-A OxCAP-MH	Baseline, weeks 12, 24, 36 and 48
To examine the health/social care and broader societal costs of patients relative to placebo over 48 weeks	Change in the following over 48 weeks: HEQ	Baseline, weeks 12, 24, 36 and 48

GAD-7, General Anxiety Disorder Scale; HEQ, Health Economics Questionnaire; ICECAP-A, ICEpop capability measure for adults; OxCAP-MH, Oxford CAPabilities questionnaire-Mental Health; QIDS-SR16, quick inventory of depressive symptomatology self-report 16; QUIP-RS, Questionnaire for Impulsive-Compulsive Disorders in Parkinson’s Disease–Rating Scale; SHAPS, Snaith-Hamilton Pleasure scale; TSQM-9, Treatment Satisfaction Questionnaire for Medication Version 9; WSAS, Work and Social Adjustment Scale.

## Methods and analysis

### Design

**Figure 1:**
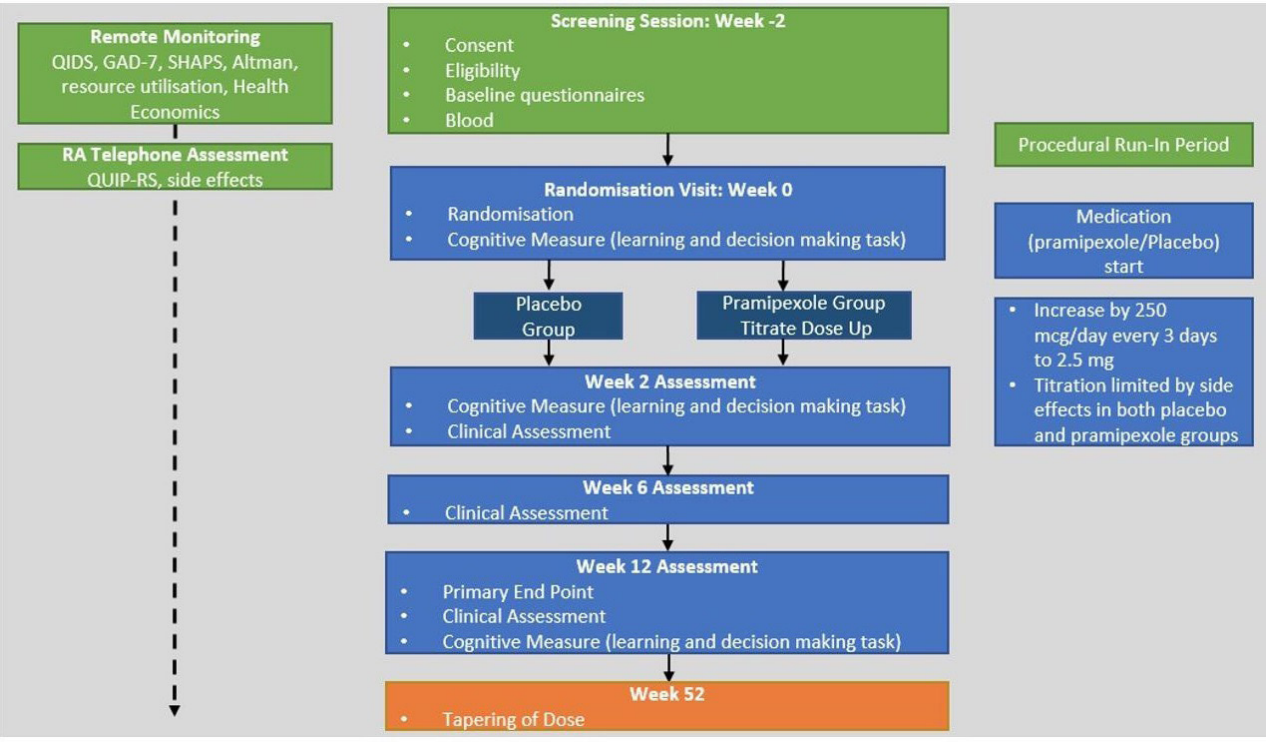
Participant timeline. GAD-7, General Anxiety Disorder Scale 7; RA, research assistant; SHAPS, Snaith-Hamilton Pleasure Scale; QIDS, quick inventory of depressive symptomatology; QUIP-RS, Questionnaire for Impulsive-Compulsive Disorders in Parkinson’s Disease-Rating Scale.

PAX-D is a multisite, double-blind, placebo-controlled, randomised trial evaluating the effects of the addition of pramipexole to antidepressant treatment in patients with TRD. Participant involvement in the trial will have two phases, a pre-treatment, pre-randomisation, run-in phase and a postrandomisation 48-week treatment phase (see figure 1). The run-in phase will assess potential participants’ ability to complete study activities and to mitigate baseline inflation effects on outcome measures. Since there is no widely accepted, first-line treatment for TRD, the comparator in the current trial will be addition of placebo. Participants will be randomised to receive either pramipexole or placebo at the randomisation visit. Participants, investigators and the trial team will remain blind to allocation. Pharmacy staff will be unblinded for dispensing purpose.

### Recruitment

Participants will be recruited from primary and secondary care services associated with the trial sites or by self-referral. The study will be advertised to local clinicians and in local and online media. Following a single-site internal pilot at the Oxford Health National Health Service (NHS) Foundation Trust, recruitment will continue in four additional sites across the UK (Newcastle, Bristol, Kings College London and University College London), and then extended to other NHS mental health trusts in regions around these sites as needed.

### Eligibility

**Figure 2:**
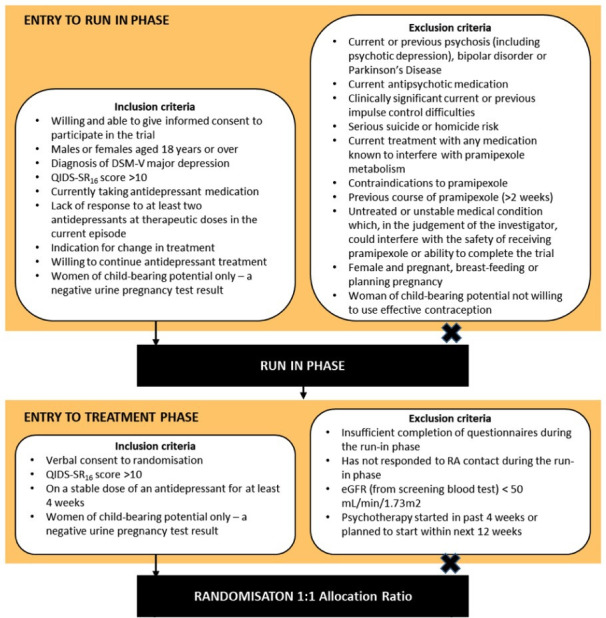
Eligibility criteria. RA, research assistant; QIDS-SR16, quick inventory of depressive symptomatology self-report.

The eligibility criteria are summarised in figure 2. During the study visits, participants will be assessed by a research assistant (RAs) and a psychiatrist.

### Interventions

In the Pramipexole for depression (PAX-D) trial, pramipexole will be added to an antidepressant that the participant is prescribed outside of the trial. The psychiatrist will be responsible for trial medication prescription. Pramipexole tablets will be taken orally. All dosages are reported as pramipexole salt (NB 1 mg of pramipexole salt is equivalent to 0.7 mg of pramipexole base). Pramipexole dihydrochloride monohydrate will be initiated at 0.25 mg/day and, in the absence of concerns about tolerability, the dose will be increased by 0.25 mg/day every 3 days towards a target dose of 2.5 mg/day (see online supplemental material for titration schedule). The target dose was selected to be at the upper end of those used in previous trials^6–10^ as the case series of TRD patients reported by Fawcett *et al* 10 indicated that a number of patients responded to higher doses. The titration schedule was also based on the Fawcett case series.^10^ The titration schedule may be amended at the discretion of the treating psychiatrist. Specifically, participants who are unable to tolerate an increased dose of pramipexole, for example, due to adverse effects, will be advised to reduce the dose to the highest tolerated. Participants will remain on this highest tolerated dose throughout the remainder of the trial. No re-titration will be attempted. Any dose reductions will be tapered every 3 days. This will reduce the risk of developing dopamine agonist withdrawal syndrome. For more information on managing adherence, discontinuing, modifying allocated interventions and concomitant treatments (see online supplemental material).

10.1136/ebmental-2021-300282.supp1 Supplementary data

#### Assignment of intervention

Each participant will be randomised at a ratio of 1:1 to either pramipexole or a matched placebo. A non-deterministic algorithm will be used to produce treatment groups balanced for important prognostic factors by minimising separately on four variables including (1) trial site, (2) age (18–50, vs >50), (3) gender (M/F) and (4) baseline quick inventory of depressive symptomatology self-report 16 (QIDS-SR16) severity (11–15 vs 16–20 vs >20).

### Outcomes

Questionnaires will be administered through a combination of participant self-reports, semi-structured interviews and completion by a psychiatrist during a clinic visit. Participants will complete questionnaires electronically using True Colours, an online platform accessed using electronic devices.^17^ Participants will be telephone contacted by an RA at screening, weekly from week 0 to 12, then 4-weekly up to week 48 to complete semi-structured interviews asking about adverse effects including any increase in impulsive behaviour or suicidality, any changes in medication and any problems with adherence.

#### Decision-making task

The task^16^ consists of 3 runs of 60 trials each (180 trials in total). On each trial, participants are presented with two abstract shapes (letters selected from the Agathodaimon font) and choose the shape which they believe will result in the best outcome. Two shapes are presented during ‘win trials’ and may result in winning either 20 or 0 points (with one shape leading to a win of 20 points on 70% of trials and the other shape on 30% of trials). A separate pair of shapes are associated with ‘loss trials’ and may result in losing 20 or 0 points (with one shape leading to a loss of 20 points on 70% of trials and the other shape leading to a loss on 30% of trials). The shapes used change in each run of the task. Participants must learn from the outcome of previous trials what they think the best shape to choose is. An increased reward sensitivity may cause participants to more consistently select the high probability rewarding outcome in the last half of each block, and is estimated by fitting a standard reinforcement learning model with free parameters for learning rates and outcome sensitivity to participant choices during the win trials. Participants will complete the task via True Colours on a computer during study visits at screening (practice), week 0 (Randomisation), week 2 and 12.

#### QIDS-SR16 and clinician-rated (QIDS-C)

This is a 16-item questionnaire that covers 9 symptoms of depression.^18^ The scale assesses severity of depression and change in depressive symptoms over time. Participants are instructed to score each item according to the description that best describes their experience over the past 7 days. Each of the symptoms is scored on a 4-point scale (0–3) giving a maximum possible score of 27 (not all items contribute to the total). The total score will detect change in depression symptom-severity, while item 12 will additionally act to detect suicidal thoughts. Clinicians will complete QIDS-C during all study visits. Meanwhile, participants will complete QIDS-SR16 on True Colours once per week from screening to Week 12, and then once every 4 weeks up to week 48.

#### Altman Self-Rating Mania Scale (ALTMAN)

This is a 5-item self-report questionnaire that assesses any change in the severity of symptoms of mania.^19^ Participants are instructed to score each item according to the description that best describes how they have been over the past 7 days. Each of the symptoms is scored on a 5-point scale (0–4) giving a maximum score of 20. Participant will complete ALTMAN on True Colours at screening, weekly from randomisation to week 12, and then 4-weekly up to week 48.

#### General Anxiety Disorder Scale-7

This seven-item self-report questionnaire screens for symptoms of anxiety and measures severity.^20^ Assessment is derived from the total score across all seven items. Participant will complete this on True Colours at screening, weekly from randomisation to week 12, and then 4 weekly up to week 48.

#### Snaith-Hamilton Pleasure Scale

This is a 14-item scale that measures anhedonia, the inability to experience pleasure.^21^ The items cover the domains of: social interaction, food and drink, sensory experience and interest/pastimes. Each item has four possible responses: strongly disagree, disagree, agree or strongly agree. Either of the ‘disagree’ responses score 1 point, and either of the ‘agree’ responses score 0 points. The final score ranges from 0 to 14, with higher scores indicating higher levels of anhedonia. Participant will complete this on True Colours at screening, 2-weekly from randomisation to week 12, and then 4-weekly up to week 48.

#### UCLA Loneliness Scale

This questionnaire comprises three questions that measure three dimensions of loneliness: relational connectedness, social connectedness and self-perceived isolation.^22^ The scale uses three response categories: ‘Hardly ever’ (scoring 3)/‘some of the time’ (scoring 2) /‘often’ (scoring 1). The scores are added together to give a total score (3 to 9). Participants will complete this scale on True Colours at week 0, 6, 12 and 48.

#### English Longitudinal Study of Ageing social isolation measure

This measure is derived from the English Longitudinal Study of Ageing, a nationally representative panel study of people aged 50 years or older living in England.^23^ It is a widely used measure of social isolation, often employed with some modifications. In PAX-D, the whole scale will be collected for the baseline measure, then for weeks 6, 12 and 48 the first three response options will be collected (3+times per week, 1–2 per week, 1–2 per month) with a change of the fourth response to ‘not in the past month’. Participants will complete this measure on True Colours at week 0, 6, 12, 48.

#### Treatment Satisfaction Questionnaire for Medication Version 9

The 9-item Treatment Satisfaction Questionnaire for Medication (TSQM)^24^ assesses patient-reported satisfaction of their medication. The domains covered are convenience, effectiveness and global satisfaction. Each item is rated on a seven or five-point scale. TSQM-9 will be completed during RA telephone contact at week 0, 6, 12, 24 and 48.

#### Questionnaire for Impulsive–Compulsive Disorders in Parkinson’s Disease-Rating Scale

The Questionnaire for Impulsive-Compulsive Disorders in Parkinson’s Disease–Rating Scale (QUIP-RS)^25^ has four primary questions (pertaining to commonly reported thoughts, urges/desires, and behaviours associated with impulse control disorders), each applied to the four impulse control disorders (compulsive gambling, buying, eating, and sexual behaviour) and three related disorders (medication use, spending and hobbyism). It uses a 5-point Likert scale (score 0–4 for each question) to gauge the frequency of behaviours. QUIP-RS will be completed at screening and then during RA contact weekly from week 0 to 12, then 4-weekly up to week 48.

#### Work and Social Adjustment Scale

This measures the impact of a respondent’s mental health difficulties on their ability to function in terms of five dimensions (work, home management, social leisure, private leisure and personal or family relationships).^26^ Severity is measured on an eight-point Likert scale (ranging from ‘not at all’ to ‘very severely’. The total Work and Social Adjustment Scale (WSAS) score is derived by adding the scores across all the items. Participants will complete WSAS on True Colours 4-weekly from week 0 to week 12, then at week 24, 36, and 48.

#### EQ-5D-5L

This is a standardised measure of health status and provides a generic measure of health-related quality of life for clinical and economic appraisal (https://euroqol.org/eq-5d-instruments/eq-5d-5l-about/). The scale has five dimensions (mobility, self-care, usual activities, pain/discomfort and anxiety/depression) and five levels for each dimension (no problems, slight problems, moderate problems, severe problems, extreme problems. A visual scale records the respondent’s self-rated health with endpoints labelled ‘the best health you can imagine’ and ‘the worst health you can imagine’. Participants will complete EQ-5D-5L on True Colours at week 0, 12, 24, 36 and 48.

#### ICEpop CAPability measure for Adults

This is a measure of capability for the adult (18+) population for use in economic evaluation.^27^ The measure covers attributes of well-being that were found to be important to adults in the UK. It has five dimensions (attachment, stability, achievement, enjoyment and autonomy) and assesses broader well-being. Participants will complete ICEpop capability measure for adults (ICECAP-A) on True Colours at week 0, 12, 24, 36 and 48.

#### Oxford CAPabilities questionnaire-Mental Health

This is a validated mental health specific capability well-being scale with 16 items.^28^ The items cover different domains of well-being (overall health, social and recreational activities, loss of sleep due to worry, friendship and support, having suitable accommodation, feeling safe, likelihood of discrimination and assault, freedom of personal and artistic expression, appreciation of nature, self-determination, and access to activities or employment), each scored on a 5-point Likert scale. Participants will complete Oxford CAPabilities questionnaire-Mental Health (OxCAP-MH) on True Colours at week 0, 12, 24, 36 and 48.

Health Economics Questionnaire (HEQ). The HEQ has specifically been developed for mental health economic evaluations and is now also complemented with a COVID-19-related module (https://zenodo.org/record/4559752). It measures health and social care resource use, medication, absenteeism from work and presenteeism as well as sociodemographic background information. Participants will complete this on True Colours at week 0, 12, 24, 36 and 48.

### Sample size

At week 12, PAX-D considers a three-point difference on QIDS-SR_16_ scores between pramipexole and placebo to be clinically important (with SD of the scores of 5.4 based on those observed in the CEQUEL trial^29^ and produces a standardised effect size of 0.56). The sample size to test differences between groups at 90% power and at a type one error rate of 5% would be 68 per group (136 total), increasing with 20% drop-out to 170 total. To test a similar sized difference in reward sensitivity and to ensure that type one error for both the depression score and the reward sensitivity tests is 0.05 or below, the sample size calculation can be determined using an alpha of 0.025. At an alpha of 0.025 and power of 90%, the required sample size would be 81 per group. Allowing for an estimated 20% lost to follow-up would then increase the sample size to 102 per group (204 total).

### Statistical methods

The primary outcome (change in QIDS-SR16 between baseline and week 12) will be analysed using a generalised linear mixed model utilising data collected at each weekly time point from randomisation and including the baseline outcome and minimisation factors as fixed effects. The model will include a random intercept for each participant to account for the repeated measures on the same participant and an interaction term for the treatment by time interaction to allow the treatment effect to differ at each time point.

Continuous secondary outcomes will be analysed using generalised linear models. The dichotomous secondary outcomes will be analysed using a logistic mixed effects regression model. These analyses will include a fixed effects randomised group and baseline level of the QIDS-SR_16_, with participants and trial site accounted for as random effects. Minimisation variables will be included as explanatory factors in the models. Mediational analysis will test whether changes in reward sensitivity mediate the effect of pramipexole on depressive symptoms. Health economic data analysis will assess group differences in quality of life, well-being, and work performance using a cost-utility analysis.

The primary and efficacy-based secondary analyses will be performed using an intention-to-treat approach for all randomised participants. Analyses of the mechanistic secondary outcomes and health economic outcomes will be performed in the set of participants who have the data required for the specific analyses (ie, no imputation will be performed for these analyses). Acceptability analyses will be performed on a subgroup of participants and trial clinicians who provide separate consent for aspect of the trial.

The main health economic analysis will include: (1) a detailed patient-level cost analysis of health, social care and other broader societal costs for both the pramipexole and placebo arms of the trial and (2) an incremental within-trial economic evaluation comparing the pramipexole and placebo arms of the trial in terms of their costs and outcomes over the 48-week trial follow-up period.

The primary health economic analysis will be a cost-utility analysis from a health and social care perspective where quality-adjusted life-years will be calculated using utility values from the EQ- 5D-5L. Secondary economic analyses using the ICECAP-A and the OxCAP-MH capability indices as outcome measures will be also carried out. Further analyses will estimate cost-effectiveness from a societal perspective. All economic analyses will be on an intention-to-treat basis.

## Discussion

PAX-D aims to test the effectiveness of pramipexole as an add-on medication to antidepressant treatment for people with TRD. Pharmacological options for patients with TRD are currently limited.^2^ The PAX-D trial is therefore timely and has the potential to inform best practice for this group of hard-to-treat patients. Pramipexole is a selective dopamine agonist and may have the potential to target symptoms of low motivation and anhedonia which are not targeted by readily available serotoninergic antidepressants. Dopamine is known to play a role in reward-based and punishment-based learning. The design of the trial also allows investigation of the mechanistic effects of pramipexole on reward sensitivity and whether changes in reward sensitivity can predict response to treatment. Furthermore, the trial will examine the acceptance, tolerability and cost-effectiveness of pramipexole treatment, and results from this study will inform clinical practice.^30^

10.1136/ebmental-2021-300282.supp2 Supplementary data

## Data Availability

No data are available. The full ethically approved protocol is available here: https://www.fundingawards.nihr.ac.uk/award/16/127/17.
