# Supplementary material for: PAX-D: study protocol for a randomised placebo-controlled trial evaluating the efficacy and mechanism of pramipexole as add-on treatment for people with treatment resistant depression
Source: Evid Based Ment Health. 2021 Nov 22;25(2):77–83. doi: 10.1136/ebmental-2021-300282 (PMC9046747; doi:10.1136/ebmental-2021-300282)
Supplement: Supplementary data [file ebmental-2021-300282supp001.pdf]

Supplemental Materials

Note. Due to limitation on space in the main manuscript, the following appendices are included to provide additional information within the Standard Protocol Items: Recommendations for Interventional Trials (SPIRIT) checklist. Codes corresponding to the SPIRIT checklist are provided in bracket {}.

Administrative information

Composition of the coordinating centre and trial steering committee {5d}. A Trial Steering Committee (TSC) will be convened to review the trial protocol, receive progress and safety reports throughout the trial and assess the implications for the trial of any new research data that becomes available. In addition to a chairperson, the committee will include independent and lay members and meetings will be attended by the CI and Trial Manager.

The trial will be supervised on a day-to-day basis by the Trial Management Group (TMG). This group reports to the TSC which is responsible to the trial sponsor. The TMG will include the CI, clinicians and experts from relevant specialties and trial staff. The TMG will review substantial amendments to the protocol prior to submission to the REC and MHRA.

A Patient Advisory Group will be established to provide insight from those with lived experience of TRD. The group will be comprised of 4 to 6 people included patients and carers. The group will help ensure the research is both relevant and feasible, and will be involved in 1) finalising trial design making sure what we are asking participants to do is reasonable, 2) ensuring recruitment and participant information is presented in a way that is understandable, 3) evaluation of the pilot phase, 4) discussing the interpretation of the data, and 5) planning the dissemination of results.

Methods: Participants, interventions, and outcomes

Intervention {11b, 11c}. Packs of trial medication will be posted to participants' nominated address. Packs will include a written schedule indicating how many tablets should be taken on each day. The dosing schedule will also be explained to patients during the randomisation visit. If subsequent dose changes need to be made the participant will be provided with a revised schedule. Participants will be asked to report adherence to trial treatment and number of tablets remaining at each study visit and each RA contact. Returned tablet counts will be used to provide a further test of adherence. The RA will inform the treating psychiatrist if patients are missing >10% of their pramipexole doses.

Participants will be asked to avoid any changes in any concomitant medication during the first 12 weeks of the randomised phase unless clinically mandatory. Participants reporting nausea after commencing pramipexole treatment may be prescribed an antiemetic that is not a centrally acting D2 antagonist. New courses of psychological therapy should not be commenced within the

first 12 weeks of randomisation. When participants reach 48-weeks post-randomisation they will attend a final trial visit where they and the investigator may discuss ongoing treatment options. For participants stopping pramipexole medication a tapering schedule will be provided to avoid withdrawal effects.

Titration schedule. See below table,

Days	Dose mg/day	Tablets
1 – 3	0.25	1 x 0.25mg
4 – 6	0.5	2 x 0.25mg
7 – 9	0.75	3 x 0.25mg
10 – 12	1.0	1 x 1.0mg
13 – 15	1.25	1 x 1.0mg + 1 x 0.25mg
16 – 18	1.5	1 x 1.0mg + 2 x 0.25mg
19 – 21	1.75	1 x 1.0mg + 3 x 0.25mg
22 – 24	2.0	2 x 1.0mg
25 – 27	2.25	2 x 1.0mg + 1 x 0.25mg
28 -	2.5	2 x 1.0mg + 2 x 0.25mg

Assignment of interventions

Sequence generation {16a}. Randomisation will be performed centrally via an online randomisation system. The first 10 participants will be allocated treatment randomly without minimisation to avoid predictability. Subsequently, the minimisation algorithm will be applied with an allocation ratio that is not fully deterministic: there will be an 80% bias in favour of allocations that minimise the imbalance. Central randomisation will aid allocation concealment (Shulz, 1995)*. *Schulz KF. Subverting Randomization in Controlled Trials. Jama-J Am Med Assoc. 1995;274(18):1456-8.

Allocation concealment mechanism {16b}. A pack ID will be generated from the online randomization system during the randomization visit. The investigator will record the Pack ID on a prescription form with the participant's details which will be sent to pharmacy for dispensing. The dispensary team will have access to an allocation list which will determine whether the participant will receive pramipexole or placebo in accordance with the pack ID assigned. Active and placebo will be packaged and labelled identically.

Unblinding {17b}. Participants will not be routinely unblinded at the end of the trial, except for an emergency or for participants wishing to continue pramipexole treatment after the end of the trial treatment period (in which case unblinding will occur after all other trial activity has

completed).

Methods: Data collection, management, and analysis

Plans to promote participant retention and complete follow-up {18b}. Participants who meet all eligibility criteria at screening will enter into the run-in phase. The minimum duration of this phase will be 1 week. Eligibility for randomisation will usually be assessed after 2 weeks but the run-in phase can be extended, for example, if antidepressant treatment has been changed. If randomisation is delayed beyond 35 days after the screening visit the participant will be withdrawn from the trial and, if appropriate, re-screened at a future date when baseline assessments will be repeated. The run-in phase is designed to minimise post-randomisation dropout by allowing assessment of symptom stability and of the likelihood that a participant will adhere to remotely recorded self-reports prior to randomisation. RAs will monitor completion of online ratings regularly and contact participants who are not adhering to the schedule.

Data management {19}. Personal data held at the Coordinating Centre will be stored securely and will only be accessible to staff who need to contact participants. Pharmacy staff will also need this information in order to dispense and mail medication to participants. Direct access to trial data, source data and medical records will be granted to authorised representatives from the Sponsor, host institution and the regulatory authorities to permit trial-related monitoring, audits and inspections. Trial data will be collected using paper or electronic case report forms (CRFs) and transferred for storage in the clinical database. All paper and electronic data will be stored securely in compliance with data protection regulations. A complete description of data management aspects of the trial is defined in the Data Management Plan.

Statistical methods to handle missing data {20c}. Missing values in all outcomes will be inspected and reported across treatment group and follow-up time. For variables measured at baseline and one follow-up interval, logistic regression will be performed to explore the effects of treatment and baseline measure on 'missingness' of each outcome. For outcome variables repeated and measured for the full 48 weeks of the follow-up period, multilevel logistic regression will be performed to examine the influence of treatment status, baseline measures and country on missingness of each outcome. The missing value pattern(s) and logistic regression results will be used to inform missing value imputation under the 'Missing At Random' assumption.

Composition of the data monitoring committee, its role and reporting structure {21a}. A Data Monitoring Committee (DMC) will be convened to review unblinded comparative data and make recommendations to the TSC on whether there are any ethical or safety reasons why the trial should not continue. The DMC will review recruitment and the interim data from the internal pilot

and convene at least annually thereafter. The DMC will be independent of the PIs and the TSC.

Interim Analysis [21b]. The internal pilot will be evaluated quantitatively and qualitatively after 6 months of recruitment at one site to address any barriers to recruitment, reported difficulties, drop-out, retention rates and reasons for discontinuation. A key question which will be addressed is whether any adjustment of the dose escalation is needed and/or whether the titration schedule should be adjusted. In addition, data quality (average response times and accuracy) from the decision-making task will be assessed to ensure that participants understand the task and are completing it appropriately.

Adverse event reporting and harms {22}. Clinically significant results will be notified to the recruiting clinician. Participants who become pregnant will be asked to consent to be followed up until the end of the pregnancy and any complications or other AE experienced by mother and/or baby reported as required. All AEs will be recorded and relationship of each AE to the trial medication must be determined by a medically qualified individual. AEs considered related to the trial medication as judged by a medically qualified investigator or the Sponsor, will be followed until resolution or until the event is considered stable.

Adverse events of special interest (AESIs) for pramipexole including 1) Impulse control disorder, and 2) Psychosis (including psychotic depression) must be reported regardless of their seriousness within 24 hours of becoming aware of the event to the Coordinating Centre. AESIs must be followed up until resolution and AESIs will be listed within the DSUR. The coordinating centre will ensure the SAE for review by the CI or a safety delegate. SAEs will also be reviewed at regular intervals by the TSC and DMC. Expectedness will be determined by the CI, or their delegate, according to the approved Reference Safety Information (SmPC) for Pramipexole. All SUSARs will be reported by the sponsor delegate to the relevant parties as applicable.

Auditing {23}. The PI will be responsible for the running of the trial at their site. This will include ensuring successful recruitment, staff education and training, and study data completeness and quality. The trial will undergo a risk assessment prior to starting, which will be reviewed at regular intervals to reflect significant changes to the protocol or outcomes of monitoring activities. A monitoring plan will be developed based on the risk assessment and will include triggers for 'for cause' site monitoring. No other routine monitoring or auditing will be conducted unless central monitoring triggers cause to do so.

Ethics and dissemination

Plans for communicating important protocol amendments to relevant parties (e.g. trial participants, ethical committees) {25}. The TMG will submit any substantial amendments to the

protocol prior to the REC and MHRA. All PIs will be kept informed of substantial amendments.

Informed Consent (26a, 26b). The investigator who obtains consent will be a suitably qualified and experienced psychiatrist, delegated to do so by the Site PI. Additional (optional) consent will be sought from participant for an additional 6ml blood sample to be taken at the randomisation visit for use in future research and for DNA extraction aimed at understanding the genetic influences on disease. During the course of the trial a participant may choose to withdraw from the trial treatment at any time. Participants may choose to stop treatment and/or study assessments but may remain on study follow-up or withdraw from the study completely. In addition, the Investigator may discontinue a participant from the trial treatment at any time if the Investigator considers it necessary for reasons including, but not limited to pregnancy, ineligibility, significant protocol deviation, significant non-compliance with treatment regimen, adverse events, disease progression, withdrawal of consent, loss to follow-up.

Confidentiality {27}. The study will comply with the General Data Protection Regulation and Data Protection Act 2018, which require data to be de-identified as soon as it is practical to do so. The processing of the personal data of participants will be minimised by making use of a unique participant study number only on all study documents and any electronic database(s), with the exception of the registration CRF, which will collect identifiers and contact details. All documents will be stored securely and only accessible by study staff and authorised personnel. The study staff will safeguard the privacy of participants' personal data.

Availability of data and materials {29, 31c}. The full study protocol is publicly available (https://fundingawards.nihr.ac.uk/award/16/127/17). De-identified individual clinical trial participant-level data will be made available for sharing in ethically approved individual patient data synthesis and meta-analyses on receipt of appropriate application and subject to approval by data sharing committee (to be convened during trial).

Post trial care {30}. As pramipexole is not licensed for use in TRD, availability outside of the study varies across the UK and would be according to the local Trust prescribing policy. For participants wanting to continue pramipexole, local provision would need to be arranged. Where a participant will continue treatment with pramipexole after the trial, the unblinding procedure will be after completion of all study activities.

Dissemination plans {31a}. The findings will be published in open-accessed peer-reviewed journals. A full account of the research will also be published in the relevant NIHR programme journal.

Appendices

Informed consent materials {32}. Informed Consent Form available on request.

Biological samples {33}. For eligible participants at randomisation, a 6ml blood sample will be taken to measure high-sensitivity C-reactive protein (hs-CRP). CRP is an inflammatory marker that may predict response to specific antidepressants (Uher et al. 2014)**. Samples may be kept for up to 1 year after the end of the trial. If optional consent has been given, an additional blood sample will be taken to be stored for future research and used for DNA extraction.

**Uher R, Tansey KE, Dew T, Maier W, Mors O, Hauser J, et al. An Inflammatory Biomarker as a Differential Predictor of Outcome of Depression Treatment With Escitalopram and Nortriptyline. Am J Psychiat. 2014;171(12):1278-86.
