# Supplementary material for: PAX-D: study protocol for a randomised placebo-controlled trial evaluating the efficacy and mechanism of pramipexole as add-on treatment for people with treatment resistant depression
Source: Evid Based Ment Health. 2021 Nov 22;25(2):77–83. doi: 10.1136/ebmental-2021-300282 (PMC9046747; doi:10.1136/ebmental-2021-300282)
Supplement: Supplementary data [file ebmental-2021-300282supp002.pdf]

Date and version No: Version 3, 24/08/2021

PAX-D

TRIAL PROTOCOL

Trial Title: Randomised placebo-controlled trial evaluating the efficacy and mechanism of pramipexole as add-on treatment for people with treatment resistant depression

Short title: PAX-D

Ethics Ref: 19/SW/0216

IRAS Project ID: 253702

EudraCT Number: 2019-001023-13

ISRCTN ISRCTN84666271

Date and Version No: Version 3, 24/08/2021

Chief Investigator: Professor Michael Browning, Department of Psychiatry

Sponsor: University of Oxford

Funder: NIHR Efficacy and Mechanism Evaluation Programme (16/127/17)

Chief Investigator Signature:

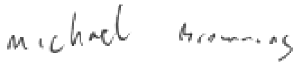

Statistician Signature:

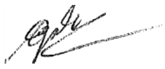

Au-Yeung SK, *et al. Evid Based Ment Health* 2022; 25:77–83. doi: 10.1136/ebmental-2021-300282

Protocol signatures continued

The Principal Investigator at each site should sign below to document that the protocol has been read and understood before the protocol is filed in the site ISF.

Trial Title: Randomised placebo-controlled trial evaluating the efficacy and mechanism of pramipexole as add-on treatment for people with treatment resistant depression (PAX-D)

EudraCT Number: 2019-001023-13

Protocol Date and Version No: Version 3, 24/08/2021

Protocol signature page

The undersigned has read and understood the trial protocol detailed above and agrees to conduct the trial in compliance with the protocol.

**Principal Investigator**<br>(Please print name)	**Site name**	**Signature**	**Date**

Date and version No: Version 3, 24/08/2021

TABLE OF CONTENTS

1 KEY TRIAL CONTACTS 7
2 LAY SUMMARY 8
3 SYNOPSIS 8
4 ABBREVIATIONS 9
5 TRIAL SCHEMA 12
6 BACKGROUND AND RATIONALE 13
6.1 Currently Available Treatment Options in Treatment Resistant Depression (TRD) 13
6.2 Pramipexole as a Novel Treatment for TRD 13
6.2.1 Current evidence for dosages of pramipexole for TRD 13
6.2.2 Justification for dosage of Pramipexole to be used in current study 14
6.3 Role of Dopamine in Reward Processing and Depression 15
6.4 Economic Analysis of Pramipexole 16
6.5 Qualitative Assessment of the Acceptability of Pramipexole 16
6.6 Mechanistic and Efficacy Hypotheses to be tested 16
7 OBJECTIVES AND OUTCOME MEASURES 17
8 TRIAL DESIGN 19
9 PARTICIPANT IDENTIFICATION 19
9.1 Trial Participants 19
9.2 Inclusion Criteria 19
9.3 Exclusion Criteria 20
10 TRIAL PROCEDURES 20
10.1 Recruitment 21
10.2 Informed Consent 21
10.3 Screening and Eligibility Assessment 22
10.3.1 Screening Visit 22
10.3.2 Baseline Assessments 22
10.4 True Colours 22
10.5 Run-in Phase 23
10.6 Randomisation 23
10.6.1 Randomisation Visit 23
10.6.2 Method of Randomisation 24
10.7 Blinding and code-breaking 24
10.7.1 Emergency Unblinding 24
10.7.2 Unblinding prior to study data lock 25

10.7.3 Effectiveness of Blinding ... 25
10.8 Subsequent Trial Visits ... 25
10.8.1 Treatment Phase Visits ... 25
10.8.2 Research Assistant Contact Between Clinic Visits ... 26
10.9 Sample Handling ... 27
10.9.1 Sample Handling for Standard of Care ... 27
10.9.2 Sample Handling for Trial Purposes ... 27
10.9.3 Sample Handling for Future Research ... 27
10.10 Early Discontinuation/Withdrawal of Participants ... 27
10.11 Definition of End of Trial ... 28
11 TRIAL INTERVENTIONS ... 28
11.1 Investigational Medicinal Product(s) (IMP) Description ... 28
11.1.1 Blinding of IMPs ... 29
11.1.2 Storage of IMP ... 29
11.1.3 Provision of trial medication to participants ... 29
11.1.4 Titration and tapering of pramipexole/placebo ... 30
11.1.5 Compliance with Trial Treatment ... 31
11.1.6 Accountability of the Trial Treatment ... 31
11.1.7 Concomitant Medication ... 31
11.1.8 Post-trial Treatment ... 31
11.2 Other Treatments (non-IMPS) ... 31
11.3 Other Interventions ... 31
11.4 Tolerability and safety ... 31
11.4.1 Side-effects of pramipexole ... 31
11.4.2 Impulse control symptoms ... 32
11.4.3 Management of Overdose ... 32
12 SAFETY REPORTING ... 32
12.1 Adverse Event Definitions ... 32
12.2 Assessment results outside of normal parameters as AEs and SAEs ... 34
12.3 Assessment of Causality ... 34
12.4 Contraception and Pregnancy ... 34
12.5 Procedures for Reporting Adverse Events ... 35
12.6 Adverse Events of Special Interest (AESI) ... 35
12.7 Reporting Procedures for Serious Adverse Events ... 36
12.7.1 Events exempt from immediate reporting as SAEs ... 38

12.7.2 Procedure for immediate reporting of Serious Adverse Events 38
12.8 Expectedness 38
12.9 SUSAR Reporting 38
12.10 Development Safety Update Reports 38
13 STATISTICS 38
13.1 Statistical Analysis Plan (SAP) 38
13.2 Description of Statistical Methods 39
13.3 Sample Size Determination 39
13.4 Analysis Populations 39
13.5 Stopping Rules 39
13.6 Procedure for Accounting for Missing, Unused, and Spurious Data 40
13.7 Health Economics Analysis 40
13.8 Internal Pilot and Evaluation of Trial Performance 41
14 DATA MANAGEMENT 42
14.1 Source Data 42
14.2 Use of Personal Data 42
14.3 Access to Data 42
14.4 Data Recording and Record Keeping 42
15 QUALITY ASSURANCE PROCEDURES 43
15.1 Risk assessment 43
15.2 Monitoring 43
15.3 Trial committees 43
15.3.1 Trial Steering Committee 43
15.3.2 Data Monitoring Committee 43
15.3.3 Trial Management Group 44
16 PROTOCOL DEVIATIONS 44
17 SERIOUS BREACHES 44
18 ETHICAL AND REGULATORY CONSIDERATIONS 44
18.1 Declaration of Helsinki 44
18.2 Guidelines for Good Clinical Practice 45
18.3 Approvals 45
18.4 Other Ethical Considerations 45
18.5 Progress Reporting 45
18.6 Participant Confidentiality 45
18.7 Expenses and Benefits 46

18.8 Peer Review 46
18.9 Patient and Public Involvement 46
18.10 Conflicts of Interest 46
19 FINANCE AND INSURANCE 46
19.1 Funding 46
19.2 Insurance 46
19.3 Contractual Arrangements 47
20 PUBLICATION POLICY 47
21 DATA SHARING 47
22 DEVELOPMENT OF A NEW PRODUCT/ PROCESS OR THE GENERATION OF INTELLECTUAL PROPERTY 47
23 ARCHIVING 47
24 REFERENCES 47
25 APPENDIX A: SCHEDULE OF PROCEDURES – CLINIC VISITS 52
26 APPENDIX B: SCHEDULE OF PROCEDURES – RA CONTACT AND SELF REPORTING 53
27 APPENDIX C: DECISION-MAKING TASK 54
28 APPENDIX D: QUESTIONNAIRES/RATING SCALES 56
29 APPENDIX E: AMENDMENT HISTORY 60

Date and version No: Version 3, 24/08/2021

1 KEY TRIAL CONTACTS

**Chief Investigator**	Dr Michael Browning<br>Department of Psychiatry<br>Warneford Hospital<br>Oxford OX3 7JX<br>01865 618 316<br>michael.browning@psych.ox.ac.uk
**PAX-D Coordinating Centre**	James Griffiths<br>Trial Manager<br>Department of Psychiatry<br>Warneford Hospital<br>Oxford OX3 7JX<br>01865 618 160<br>pax-d@psych.ox.ac.uk
**Sponsor**	University of Oxford<br>Joint Research Office<br>1st floor, Boundary Brook House<br>Churchill Drive, Headington<br>Oxford OX3 7LQ
**Funder(s)**	NIHR Efficacy and Mechanism Evaluation Programme
**Statistician**	Ushma Galal<br>Medical Statistician<br>Nuffield Department of Primary Care Health Sciences<br>Radcliffe Observatory Quarter, Woodstock Road<br>Oxford. OX2 6GG<br>01865 617 878<br>ushma.galal@phc.ox.ac.uk
**Oversight Committees**	Trial Steering Committee Chair:<br>Rachel Upthegrove<br>School of Psychology<br>University of Birmingham<br>Edgbaston, Birmingham, B15 2TT<br>0121 414 6241<br>r.upthegrove@bham.ac.uk<br><br>Data Monitoring Committee Chair:<br>Professor Bill Deakin<br>Division of Neuroscience & Experimental Psychology<br>University of Manchester<br>Oxford Road<br>Manchester M13 9PL<br>bill.deakin@manchester.ac.uk

Au-Yeung SK, *et al. Evid Based Ment Health* 2022; 25:77–83. doi: 10.1136/ebmental-2021-300282

2 LAY SUMMARY

Clinical depression is a common disorder usually treated in primary care with psychological therapies and antidepressant medication. However, a significant proportion of people (about 2-3 in 10) do not improve with current first-line therapies and are regarded as having treatment resistant depression (TRD).

TRD is a major problem for both patients and society because of the high level of suffering and associated disability. Current medicines for TRD are not particularly effective for many people and often have adverse effects which patients find distressing.

There is some evidence that pramipexole, a medicine already commonly used in Parkinson's Disease, may be an effective treatment for TRD. PAX-D will compare the effects of pramipexole with placebo when added to current antidepressant medication for people with TRD. The trial will look at effectiveness in the short- term (after 12 week's treatment) and in the longer-term (48 weeks). The trial will also assess the adverse effects of pramipexole and explore patients' experiences of taking it.

Pramipexole is unlike current antidepressant drugs in that it acts on a brain chemical called dopamine, which is known to influence people's motivation to pursue goals and affect how rewarding they find them. Lack of motivation is a key symptom of depression so any antidepressant effects of pramipexole may be linked to increased motivation. PAX-D participants will be asked to carry out a computer task designed to measure how pramipexole affects the dopamine system in the brain and how far this can explain its antidepressant effects.

If pramipexole is effective it could become a very useful treatment option for patients with TRD and this information will be disseminated through scientific publications, meetings with patient groups and NHS innovation programmes.

3 SYNOPSIS

Trial Title	Randomised placebo-controlled trial evaluating the efficacy and mechanism of pramipexole as add-on treatment for people with treatment resistant depression
Short title	PAX-D
Trial registration	ISRCTN84666271
Sponsor	University of Oxford
Funder	NIHR Efficacy and Mechanism Evaluation Programme
Clinical Phase	4
Trial Design	Randomised, double-blind, placebo-controlled trial
Trial Participants	Adults aged 18 years or older diagnosed with treatment resistant depression
Sample Size	204 (including internal pilot)
Planned Trial Period	Overall, including set-up the trial is expected to last four years (from April 2019 to March 2023).

Date and version No: Version 3, 24/08/2021

Planned Recruitment period	November 2019 to December 2021		
	Objectives	Outcome Measures	Timepoint(s)
Primary	To compare the efficacy of pramipexole and placebo at 12 weeks post-randomisation	Improvement (change from baseline) of depressive symptoms measured on the Quick Inventory of Depressive Symptomatology, self-report version (QIDS-SR_{16}).	Baseline, Week 12
Secondary	For details of secondary objectives and outcome measures see section 7.		
IMP(s)	Pramipexole<br>Tablets to be taken orally. Pramipexole dihydrochloride monohydrate will be initiated at 0.25mg/day and, in the absence of concerns about tolerability, the dose will be increased by 0.25mg/day every three days towards a target dose of 2.5mg/day. Participants unable to tolerate a scheduled dose increase will be advised to remain on the highest tolerable dose.		
NIMP(s)	Patient's standard antidepressant medication		
Comparator	Placebo matched to pramipexole tablets		

4 ABBREVIATIONS

AE	Adverse event
ALTMAN	Altman Self Rating Scale for Mania
AR	Adverse reaction
CPSU	Oxford Health NHS Foundation Trust Clinical Pharmacy Support Unit
CI	Chief Investigator
CRF	Case Report Form
CRN	Clinical Research Networks
DMC	Data Monitoring Committee
DSUR	Development Safety Update Report
ECT	Electroconvulsive Therapy
ELSA	English Longitudinal Study of Aging
EQ-5D-5L	5-level EQ-5D version
GAD-7	Generalised Anxiety Disorder Scale
GCP	Good Clinical Practice
GP	General Practitioner

Au-Yeung SK, *et al. Evid Based Ment Health* 2022; 25:77–83. doi: 10.1136/ebmental-2021-300282

HDPE	High-density Polyethylene
HEQ	Health Economics Questionnaire
Hs-CRP	High sensitivity C-Reactive Protein blood test
ICECAP-A	ICEpop CAPability measure for Adults
ICF	Informed Consent Form
ICH	International Conference of Harmonisation
IMP	Investigational Medicinal Product
LVLP	Last Visit Last Patient
MHRA	Medicines and Healthcare products Regulatory Agency
MINI	The Mini International Neuropsychiatric Interview
NHS	National Health Service
NIHR	National Institute for Health Research
NIHR-CRF	NIHR cognitive health Clinical Research Facility
NRES	National Research Ethics Service
OxCAP-MH	Oxford CAPabilities questionnaire-Mental Health
PI	Principal Investigator
PIS	Participant Information Sheet
PPI	Patient and Public Involvement
QALYs	Quality-adjusted life years
QIDS-C	Quick Inventory of Depressive Symptomatology – clinician rated version
QIDS-SR_{16}	Quick Inventory of Depressive Symptomatology – self report version
QUIP-RS	Questionnaire for Impulsive-Compulsive Disorders in Parkinson's Disease– Rating Scale
RA	Research Assistant
R&D	NHS Trust R&D Department
RCT	Randomised controlled trial
REC	Research Ethics Committee
SAE	Serious Adverse Event
SAP	Statistical Analysis Plan
SAR	Serious Adverse Reaction
SDV	Source Data Verification
SHAPS	Snaith-Hamilton pleasure scale
SmPC	Summary of Product Characteristics
SOP	Standard Operating Procedure
SUSAR	Suspected Unexpected Serious Adverse Reactions

Au-Yeung SK, *et al. Evid Based Ment Health* 2022; 25:77–83. doi: 10.1136/ebmental-2021-300282

TC	True Colours
TCA	Tricyclic antidepressant
TMF	Trial Master File
TRD	Treatment Resistant Depression
TSC	Trial Steering Committee
TSQM	Treatment Satisfaction Questionnaire for Medication
U&Es	Urea and electrolytes blood test
WOCBP	Woman of child-bearing potential
WSAS	Work and Social Adjustment Scale

Au-Yeung SK, *et al. Evid Based Ment Health* 2022; 25:77–83. doi: 10.1136/ebmental-2021-300282

Date and version No: Version 3, 24/08/2021

5 TRIAL SCHEMA

ENTRY TO RUN IN PHASE

Inclusion criteria

- Willing and able to give informed consent to participate in the trial
- Males or females aged 18 years or over
- Diagnosis of DSM-V major depression
- QIDS-SR_{16} score >10
- Currently taking antidepressant medication
- Lack of response to at least two antidepressants at therapeutic doses in the current episode
- Indication for change in treatment
- Willing to continue antidepressant treatment
- Women of child-bearing potential only – a negative urine pregnancy test result

Exclusion criteria

- Current or previous psychosis (including psychotic depression), bipolar disorder or Parkinson's Disease
- Current antipsychotic medication
- Clinically significant current or previous impulse control difficulties
- Serious suicide or homicide risk
- Current treatment with any medication known to interfere with pramipexole metabolism
- Contraindications to pramipexole
- Previous course of pramipexole (>2 weeks)
- Untreated or unstable medical condition which, in the judgement of the investigator, could interfere with the safety of receiving pramipexole or ability to complete the trial
- Female and pregnant, breast-feeding or planning pregnancy
- Woman of child-bearing potential not willing to use effective contraception

RUN IN PHASE

ENTRY TO TREATMENT PHASE

Inclusion criteria

- Verbal consent to randomisation
- QIDS-SR_{16} score >10
- On a stable dose of an antidepressant for at least 4 weeks
- Women of child-bearing potential only – a negative urine pregnancy test result

Exclusion criteria

- Insufficient completion of questionnaires during the run-in phase
- Has not responded to RA contact during the run-in phase
- eGFR (from screening blood test) < 50 mL/min/1.73m2
- Psychotherapy started in past 4 weeks or planned to start within next 12 weeks

RANDOMISATON 1:1 Allocation Ratio

Pramipexole + Participant's Standard Antidepressant Treatment	Matched Placebo + Participant's Standard Antidepressant Treatment

Primary Outcome
Improvement of depressive symptoms at 12 weeks measured on the QIDS-SR_{16}

Secondary Outcomes
See section 7 for details

Au-Yeung SK, *et al. Evid Based Ment Health* 2022; 25:77–83. doi: 10.1136/ebmental-2021-300282

6 BACKGROUND AND RATIONALE

Depression that fails to respond to standard treatments (which is experienced by 20-30% of all depressed patients) is a leading cause of morbidity and work days lost (Fostick et al., 2010). Currently there is a shortage of effective pharmacological options for patients whose depression fails to respond to two separate courses of antidepressant medication and are therefore considered 'treatment resistant' by conventional classifications (NICE, 2009a).

6.1 Currently Available Treatment Options in Treatment Resistant Depression (TRD)

The large, pragmatic STAR*D study suggested that the chances of remission with conventional pharmacological approaches for patients with treatment resistant depression are less than 15% (Rush et al., 2006). A systematic review (Zhou et al., 2015) indicated that, at this point, the best evidence-based treatment is addition of atypical antipsychotic medications such as aripiprazole and quetiapine; however these agents are only moderately effective and have high dropout rates because of adverse events. The extent and frequency of these adverse events (sedation, weight gain and movement disorders) means that this 'atypical augmentation' is widely disliked by patients. Another option is lithium addition but the evidence base is small and lithium is poorly tolerated, potentially toxic and again disliked by patients. No new antidepressant is more effective than tricyclic antidepressants (TCAs) discovered by chance more than 60 years ago and industry has largely withdrawn from this area because of the difficulty of making real advances.

6.2 Pramipexole as a Novel Treatment for TRD

There is preliminary evidence that the dopamine agonist pramipexole could represent an important advance in TRD. The dosage schedule for the PAX-D trial is based on the SmPC for pramipexole, the 5 small RCTs of pramipexole in unipolar and bipolar depression, and a recent, large published case series of patients with TRD treated with pramipexole (Fawcett et al., 2016).

6.2.1 Current evidence for dosages of pramipexole for TRD

The SmPC for Pramipexole (doses are given as salt rather than base) for the treatment of Parkinson's disease recommends a schedule starting with a dose of 0.375mg/day (taken as 0.125mg three times daily), increasing by 0.375mg/day after 5-7 days, then in 0.75mg/day steps every 5-7 days. The recommended maximum is 4.5mg/day.

A single randomised controlled trial (RCT) of pramipexole in 65 patients with unipolar TRD has been reported (Cusin et al., 2013). In this study there was significant benefit of pramipexole over placebo in a continuous outcome measure but not in terms of response (40% versus 27%). The relatively modest outcome in this study could have been due to the lower average dose of pramipexole used (1.3 mg/day).

Another single RCT has examined pramipexole monotherapy in non-treatment resistant unipolar depression (Corrigan et al., 2000). This study, which used 3 daily doses of the drug (0.375mg, 1mg and 5mg), found that 0.375mg was ineffective, 1mg was superior to placebo and 5mg caused very high treatment drop out.

A small study (n=13 per group) of unipolar patients resistant to a single previous antidepressant were randomised to receive Pramipexole monotherapy, Pramipexole combined with escitalopram or

escitalopram monotherapy (Franco-Chaves et al., 2013). The target dose for this study was 2.25mg/day. No statistical difference between the study groups was observed, although very few patients in the combined group completed the study (n=4).

The most compelling evidence rests on a case-series of TRD patients treated in the United States (Fawcett et al., 2016). This reported very good therapeutic responses to pramipexole augmentation of antidepressant therapy in 42 patients with depression, highly refractory to conventional treatments (including ECT). In this study pramipexole was given once daily at a mean dose of around 2.5 mg/day.

The Zarate et al. (2004) RCT treated patients with bipolar depression using a more conservative dosage schedule than the SmPC, limiting all increases to 0.375mg every 5-7 days. In this study, 10 patients were treated with pramipexole with an average dose of 1.7mg. The Goldberg et al. (2004) RCT also in bipolar depression utilised smaller dosage steps of 0.25mg (0.125mg twice a day) but occurring more frequently, every 3-5 days. The average dose of pramipexole used was identical to that in the Zarate et al. study (1.7 mg/day).

The table below compares the dosage schedules from the sources described above and illustrates the potential dose (dependent on tolerability) achieved at 14 and 28 days of treatment. Ranges in doses reflect the range in frequency of dose increments used.

**Source**	**Dose after 14 days**	**Dose after 28 days**	**Target**	**Maximum**	**Average**
	**mg/day**	**mg/day**	**mg/day**	**mg/day**	**mg/day**
SmPC for Pramipexole	0.75 to 1.5	2.25 to 3.75	-	4.5	-
Cusin et al., 2013	1.5	3	3	3	1.35 (n=22)
Zarate et al., 2004	0.75 to 1.125	1.5 to 2.0	-	4.5	1.7 (n=10)
Goldberg et al., 2004	0.75 to 1.25	1.5 to 2.5	2.5	5	1.7 (n=12)
Corrigan et al., 2000	0.375 to 5.0	0.375 to 5.0	0.375, 1, 5	5	-
Franco-Chaves et al., 2013	0.75	2.25	2.25	2.25	-
Fawcett et al., 2016	Under 45s: 1.25 Over 45s: 2.5	Under 45s: 2.5 Over 45s: 5.0	2	5	2.46 (n=42)

A number of other studies (reviewed in Aiken, 2007) have also reported a beneficial effect of pramipexole in patients given pramipexole treatment for other indications. A recent systematic review (Tundo et al., 2019) identified five completed randomised controlled trials, three open label trials and five observational studies on pramipexole for patients with major depressive episodes. It concludes that adequately powered randomised controlled trials of pramipexole for major depressive episodes are needed.

6.2.2 Justification for dosage of Pramipexole to be used in current study

PAX-D will build on this prior work by assessing the efficacy of pramipexole augmentation of conventional antidepressant medication. The dosage schedule for PAX-D (see section 11.1.4) is based on that reported

by Fawcett et al., (2016). The schedule used for patients under 45 years old was chosen (0.25mg/day incremental increase every three days). This leads to doses at 14 and 28 days that are within the range that would achieved if using the schedule included in the SmPC for Parkinson's disease. The maximum dose in the PAX-D study (2.5mg/day) is lower than in two previous RCTs (Zarate et al., 2004; Goldberg et al., 2004) but higher than the actual average achieved in these two studies and the Fawcett et al. case series. The faster titration rate used by Fawcett et al. for patients over 45 was not used to simplify prescribing across a large RCT and because the slower titration rate still allows all participants to reach a dose above the average used in other studies.

Pramipexole will be administered under double-blind, controlled conditions in NHS patients with treatment resistant depression. Depression will be measured using the Quick Inventory of Depressive Symptomatology (QIDS; Rush, 2003). This rating scale has clinician-rated and self-report versions and has been used in other trials to measure the primary outcome (e.g. Geddes et al., 2016). Since there is no standard treatment for TRD, the comparator in the current trial will be addition of placebo.

6.3 Role of Dopamine in Reward Processing and Depression

Pramipexole is also of high scientific and clinical interest because it is a selective dopamine D2/D3 receptor agonist and therefore pharmacologically distinct from currently available antidepressants, most of which act to increase the level of serotonin in the synapse. Dysfunction in dopamine pathways is believed to underpin some of the most critical symptoms of severe depression, in particular the experience of low motivation and anhedonia, symptoms that are not targeted by current treatments (Shelton & Tomarken, 2001). The role of dopamine neurons in adaptive behaviours has been successfully described by a number of neurocomputational models which emphasise the role of dopamine pathways in reward and punishment-based learning, processes that are clearly relevant to patients with severe depression (Cools, 2008; Schultz et al., 1997). These computational models offer exciting possibilities in terms of 1) tracking the effects of pramipexole on reward sensitivity (a measure of the impact that a given reward has on learning and choice behaviour) over time in depressed patients; 2) assessing how far such an action contributes to its antidepressant effect; and 3) predicting at baseline which depressed patients are most likely to respond to dopamine agonist treatment. Previous studies of pramipexole in which patients with Parkinson's disease (Cools, 2006), bipolar disorder (Burdick, 2014) and cocaine abuse (Newton, 2015) completed reward learning tasks indicate that the medication acted to increase reward sensitivity.

A recently described learning and decision making task is able to capture changes in sensitivity to both rewards (winning points) and losses (losing points) simultaneously (Pulcu & Browning, 2017a). In this task sensitivity to reward is measured as a parameter of a formal model which is fitted to participant choice data. Using a computational model allows the identification and precise measurement of cognitive processes, such as reward sensitivity, which are poorly assessed using traditional techniques (Pulcu & Browning, 2017b). The model consists of a Rescorla-Wagner learner coupled to a soft-max action selector (Browning et al., 2015) with separate learning rate and decision parameters for positive and negative outcomes. The rationale for using this task in the current trial is that it requires participants to learn independently from wins (i.e. rewards) and losses (i.e. punishments) and so is able to assess both general deficits in learning and decision making, as well as specific changes in learning and decision making in response to rewarding relative to loss outcomes (and pramipexole is proposed to specifically alter reward sensitivity). The advantage of using the specific reinforcement learning task proposed for this trial is that it allows a simple assessment of reward relative to loss sensitivity in a single task block, as opposed to requiring participants to complete multiple blocks/tasks (as in Cools, 2006), or simply focussing on rewards

(as in Burdick, 2014; Newton, 2015). The task is therefore able to capture the specific mechanistic process proposed to underlie the efficacy of pramipexole and it is feasible to administer the task using an online system to participants in a multi-site trial. Furthermore, because it is both simple and online, the task could, if it proves informative, be used as a clinical tool in patients with treatment resistant depression.

This task and model will be used in the current trial to measure the impact of pramipexole on reward sensitivity enabling assessment of the role of these computationally defined processes in the mechanism of action of the drug. The relationship between pramipexole treatment, change in reward sensitivity and symptomatic response will be formally assessed using a mediation analysis. Further, the degree to which baseline and initial changes in the computational parameters are able to predict response to pramipexole will be assessed, providing a first test of the potential for these measures to be deployed in the selection of treatments for TRD patients.

6.4 Economic Analysis of Pramipexole

Treatment resistant depression presents a particular challenge to mental health services, with the specialist services designed for such patients often providing poor value for money (Morriss et al., 2016). A critical aspect of the feasibility of using pramipexole in patients with treatment resistant depression is therefore the impact of the treatment on longer term quality of life and functioning. This will be assessed in the current trial using a prospective, within trial economic analysis of the use of pramipexole over the course of 48 weeks following randomisation. Main outcomes will be expressed in terms of quality-adjusted life years (QALYs) derived from the EQ-5D-5L (EuroQol Group, 1990). Broader well-being outcome measures will be based on the capability approach using the OxCAP-MH (Simon et al., 2013) and ICECAP-A (Mitchell, 2017).

6.5 Qualitative Assessment of the Acceptability of Pramipexole

The acceptability and tolerability of treatment with pramipexole will be assessed using qualitative data purposively sampled from 10 trial patients and 5 clinicians after trial completion. This will supplement the quantitative analysis of tolerability obtained during the randomised phase of the trial and will provide an indicative assessment of patient and investigator experience of using the treatment.

6.6 Mechanistic and Efficacy Hypotheses to be tested

Hypotheses related to clinical effects:

- Primary hypothesis: That pramipexole will produce a greater improvement than placebo in depressive symptoms after twelve weeks of treatment
- That the dropout rate due to intolerance in the pramipexole arm will be less than 20% after twelve weeks of treatment
- That the rate of significant behavioural disturbance will be less than 5% after twelve weeks of treatment
- That pramipexole will improve symptoms of depression, anhedonia and anxiety as well as functional outcomes, to a greater degree than placebo, across the 48 weeks of the trial

Hypothesis related to mechanism:

- That, after two weeks treatment, pramipexole will increase sensitivity to reward relative to placebo

Hypotheses related to predictive early measurements:

- That the extent to which pramipexole increases reward sensitivity at two weeks will mediate therapeutic outcome to pramipexole at twelve weeks
- That greater impairment in reward sensitivity at baseline will predict a greater therapeutic response to pramipexole at twelve weeks
- That patients with greater degrees of anhedonia at baseline will have better therapeutic outcomes with pramipexole at twelve weeks

7 OBJECTIVES AND OUTCOME MEASURES

	Objectives	Outcome Measures	Timepoint(s)
	**Primary Objective**	**Primary Outcome**	
1	To compare the efficacy of pramipexole and placebo at 12 weeks post-randomisation	Improvement (change from baseline) of depressive symptoms measured on the Quick Inventory of Depressive Symptomatology, self-report version (QIDS-SR_{16}).	Baseline, Week 12
	**Secondary Objectives**	**Secondary Outcomes**	
1	To compare the tolerability and safety of pramipexole and placebo during the 48 week treatment phase	Tolerability assessed by:<br>• Termination of trial treatment due to intolerance<br>• Adverse reactions<br>• TSQM<br>Safety – emergence of new symptoms:<br>• ALTMAN (manic symptoms)<br>• QUIP-RS (impulse control)<br>• Suicidal ideation (QIDS-SR_{16})	Weeks 1-48
2	To compare the effect of pramipexole and placebo on reward sensitivity	Change in reward sensitivity parameter from model fitted to learning/decision making task between baseline, week 2 and week 12	Baseline, Week 2, Week 12
3	To test the degree to which change in reward sensitivity mediates the 12 week response to pramipexole of	Change in QIDS-SR_{16} and SHAPS scores between baseline and week 12 and change in	Baseline, Week 2, Week 12

	both depressive and specifically anhedonic symptoms	reward sensitivity between baseline and week 2	
4	To compare the extent to which an increase in reward sensitivity predicts therapeutic response	Change scores in the learning/decision making task at 2 weeks and the change in the QID-SR_{16} at 12 weeks	Week 2, Week 12
5	To explore the extent to which reward sensitivity at baseline predicts therapeutic response	Baseline scores on the learning/decision making task and the change in QIDS-SR_{16} at 12 weeks	Baseline, Week 12
6	To explore the extent to which level of anhedonia at baseline predicts therapeutic response	Baseline scores on the SHAPS and change in the QID-SR_{16} at 12 weeks	Baseline, Week 12
7	To compare the effect of pramipexole and placebo on the trajectory of symptoms of depression	QIDS-SR_{16} scores collected over 48 weeks of the trial	Weeks 1-48
8	To compare the effect of pramipexole and placebo on response and remission rates, using the QIDS-SR_{16}, at twelve weeks	QIDS-SR_{16} response, defined as a reduction of ≥50% in baseline score by week 12. QIDS-SR_{16} remission defined as a score of ≤5 at week 12	Weekly for weeks 1-12
9	To compare the impact of pramipexole and placebo on symptoms of anhedonia, anxiety and clinician rated depression	Change scores for the SHAPS, GAD-7 and QIDS-C between baseline and week 12	Baseline, Week 12
10	To compare the impact of pramipexole and placebo on functional outcome over the 48 weeks of treatment	Change scores for the WSAS-screener between baseline and week 48	Baseline, Week 48
11	To determine the impact on quality of life and wellbeing of pramipexole relative to placebo over 48 weeks	Change in the following over 48 weeks:<br>• EQ-5D-5L<br>• ICECAP-A<br>• OxCAP-MH	Baseline, Weeks 12, 24, 36 and 48
12	To examine the health / social care and broader societal	Change in the following over 48 weeks:<br>• HEQ	Baseline, Weeks 12, 24, 36 and 48

	costs of patients relative to placebo over 48 weeks		

8 TRIAL DESIGN

PAX-D is a multi-site, double-blind, placebo-controlled, randomised trial evaluating the effects of the addition of pramipexole to antidepressant treatment in patients with TRD. Following a single site internal pilot, recruitment will continue in five NHS Trusts linked with Biomedical Research Centres which have psychiatrists who have expertise in TRD. Recruitment will then be extended to other NHS Mental Health Trusts in the regions around these sites as needed to ensure an adequate recruitment rate. Participant involvement in the trial will have two phases, a pre-treatment run-in phase and a 48-week treatment phase.

9 PARTICIPANT IDENTIFICATION

9.1 Trial Participants

Participants will be adult males or females, aged 18 years or older, with TRD who have been referred or have self-referred to one of the participating Trial Sites. The Clinical Research Network (CRN) will assist with publicity of the trial and with identification of potential patients. The trial team will work closely with the CRN Clinical Studies Officers (CSOs) who have an awareness of local clinical streams and structures, and how best to identify and access patients.

9.2 Inclusion Criteria

For entry into the run-in phase:

- Willing and able to give informed consent to participate in the trial
- Males or females aged 18 years or over
- Diagnosis of DSM-V major depression based on the affective disorder sections of the Mini International Neuropsychiatric Interview (MINI). *N.b. comorbid anxiety disorder is not an exclusion criterion*
- Quick Inventory of Depressive Symptomatology self-report version (QIDS-SR_{16}) score >10 (moderate, severe or very severe depression)
- Currently taking an antidepressant medication
- A lack of response to at least two antidepressants at therapeutic doses (based on Maudsley Prescribing Guidelines and/or British National Formulary) in the current episode
- An indication for a change in treatment
- Willing to continue antidepressant treatment
- Women of child-bearing potential (WOCBP) only – a negative urine pregnancy test result

At randomisation:

- Verbal consent to randomisation
- QIDS-SR_{16} score >10
- On a stable dose of an antidepressant for at least 4 weeks
- WOCBP only – a negative urine pregnancy test result

9.3 Exclusion Criteria

The participant may not enter the run-in phase if ANY of the following apply:

- Clinical diagnosis of current or previous psychosis (including psychotic depression), bipolar disorder or Parkinson's Disease
- Currently taking an antipsychotic medication
- Clinically significant current or previous impulse control difficulties
- Serious suicide or homicide risk
- Current treatment with any medication known to interfere with pramipexole metabolism including cimetidine, memantine and methyldopa
- Contraindications to pramipexole including history of or current treatment for eye disease (excluding near or long-sightedness), significant, symptomatic cardiovascular or renal disease or significant, symptomatic orthostatic hypotension
- Previous course of pramipexole (>2 weeks)
- Untreated or unstable medical condition which, in the judgement of the investigator, could interfere with the safety of receiving pramipexole or ability to complete the trial
- Female and pregnant, breast-feeding or planning pregnancy
- WOCBP not willing to use effective contraception

The participant may not enter the randomised phase if ANY of the following apply:

- Insufficient completion of questionnaires scheduled during the run-in phase (defined as at least 75% completion)
- Has not responded to RA contact during the run-in phase
- eGFR (from screening blood test) < 50 mL/min/1.73m^2
- Psychotherapy started in past 4 weeks or planned to start within next 12 weeks

For the purposes of eligibility assessment, a WOCBP is a sexually mature woman (i.e. any female who has experienced menstrual bleeding) who:

- Has not undergone a hysterectomy or bilateral oophorectomy/salpingectomy
- Is not postmenopausal (a post-menopausal woman is a female who has not had menses at any time in the preceding 12 consecutive months without an alternative medical cause)
- Has not had premature ovarian failure confirmed by a gynaecologist

10 TRIAL PROCEDURES

For a schedule of procedures, see Appendices B and C.

10.1 Recruitment

Recruitment to PAX-D will begin in the Oxford Health NHS Foundation Trust Treatment Resistant Depression Clinic as part of a single-centre internal pilot lasting six months (see section 13.8) for detailed description of the internal pilot). The trial will then be rolled-out to additional NHS Trusts with specialist services for TRD and subsequently to NHS Trusts across the UK.

Participants will be recruited from primary and secondary care services associated with the trial sites or by self-referral. Information about the trial design and the entry criteria will be advertised to local clinicians and in local and online (social) media. Patients who are referred or who contact the Trial Sites will be provided with information about the trial by the research team either by phone or email and, if appropriate, offered a face-to-face visit with an investigator who may invite them to consider taking part in the trial. General Practice (GP) surgeries may, with appropriate approvals, act as participant identification centres (PIC) for each trial site. Potentially eligible participants will be identified from PICs using searches of electronic health records. The PICs will then send invitation letters to potentially eligible participants, informing them of the trial and inviting them to contact the Trial Site should they be interested in participating.

Potential participants may also be identified by vendors providing specialist recruitment services, such as Lindus Health (https://www.lindushealth.com). Paid advertising will be placed on social media services (such as Facebook) and via GP partnerships. People who respond to the advert or message from their GP will be directed to the overview page on a website where they can choose to start a pre-screening questionnaire, which will ask questions about their eligibility for the trial. If the result is that they are not eligible, no data is stored. If they are eligible, then consent is obtained via the website to collect contact details, which would be passed to the study team using an appropriate encrypted method.

10.2 Informed Consent

Investigators seeing participants referred to a trial site will identify those who meet the broad eligibility criteria and are willing to consider taking part in the trial. The investigator will give the patient a copy of the current approved Participant Information Sheet (PIS) and, when appropriate, obtain Informed Consent. It is the responsibility of the investigator taking consent to assess a participant’s capacity to give informed consent.

Written and verbal versions of the PIS and Informed Consent Form (ICF) will be presented to the patient detailing the exact nature of the trial, what it would involve, the implications and constraints of the protocol, the known adverse effects and any risks involved in taking part. It will be clearly stated that the patient would be free to withdraw from the trial at any time for any reason without prejudice to future care, and with no obligation to give the reason for withdrawal. The discussion and consent process should be documented in the participant’s medical record.

Patients will be allowed as much time as they wish to consider the information and be given the opportunity to question the investigator, research staff, their GP or other independent parties to decide whether they will participate in the trial. Written Informed Consent (using the latest approved version of the ICF) will then be obtained by means of participant dated signature and dated signature of the investigator who presented the trial and obtained the Informed Consent.

The investigator who obtains the consent will be a suitably qualified and experienced psychiatrist, delegated to do so by the Site PI. A copy of the signed ICF will be given to the participant. The original

signed form will be retained in the Investigator Site File; a copy will be sent to the PAX-D Coordinating Centre and a copy placed in the participant's medical records.

Before any trial specific procedures are performed the participant must personally sign and date the latest approved version of the Informed Consent Form.

10.3 Screening and Eligibility Assessment

10.3.1 Screening Visit

Participants who give Informed Consent will attend a screening visit which may take place immediately after consent has been given or at a later date. During this visit:

- Eligibility will be reviewed in a clinical interview including diagnosis, severity (QIDS-SR_{16} and QIDS-C), duration of current depressive episode, treatment and response to treatment for this episode and for previous episodes
- A medical and psychiatric history will be taken including age of first depressive episode, number of previous episodes, other psychiatric diagnoses, family history of mood disorder, substance use and suicide/self-harm/homicide risk
- Details of any concurrent medical conditions will be recorded and height, weight, blood pressure and pulse will be measured

Eligibility must be assessed by a medically qualified doctor and this assessment documented in the participant's medical notes. Only personnel formally delegated by the Principal Investigator to assess eligibility may perform this task.

There will be no exception to the eligibility criteria. Queries in relation to the eligibility criteria must be addressed prior to entry into the run-in phase or randomisation, as appropriate. Participants are only eligible for the trial if all the inclusion criteria are met and none of the exclusion criteria apply. Protocol waivers are not permitted.

10.3.2 Baseline Assessments

Patients who meet eligibility criteria and give informed consent will undergo baseline assessments which will include recording demographic details and registration on the True Colours system (see section 10.4). The Questionnaire for Impulsive-Compulsive Disorders in Parkinson's Disease–Rating Scale (QUIP-RS: Weintraub et al., 2012) will also be completed and participants will be given the opportunity to try the decision making task which they will be asked to complete at the Randomisation, Week 2 and Week 12 visits.

Blood sample(s) will be taken for measurement of serum creatinine and calculation of eGFR, and any other pre-treatment tests that the clinician considers necessary.

10.4 True Colours

True Colours uses mobile phone and web-based technology to collect self-reported health measures. It was initially developed as a clinical tool that enabled patients with bipolar disorder to report both

Au-Yeung SK, *et al. Evid Based Ment Health* 2022; 25:77–83. doi: 10.1136/ebmental-2021-300282

depressive and manic symptoms in a format that could be displayed on graphs and viewed online both by themselves and with their permission, by members of their care team, family and/or friends.

Participants will be registered on True Colours at the Screening Visit and asked to begin completing self-ratings as prompted. At the randomisation visit the participants will be set up to receive additional questionnaires at varying intervals according to the table overleaf.

Self-reporting online using True Colours			
	**Initiation of scale**	**Weeks 0 – 12**	**Weeks 13 - 48**
QIDS-SR$_{16}$	Screening	✓ Weekly	✓ 4-weekly
ALTMAN	Screening	✓ Weekly	✓ 4-weekly
GAD-7	Screening	✓ Weekly	✓ 4-weekly
SHAPS	Screening	✓ 2- weekly	✓ 4-weekly
UCLA Loneliness	Randomisation	✓ Weeks 6, 12	✓ Week 48
ELSA Social Isolation	Randomisation	✓ Weeks 6, 12	✓ Week 48
WSAS	Randomisation	✓ 4-weekly	✓ Weeks 24, 36 and 48
EQ-5D-5L	Randomisation	✓ Week 12	✓ Weeks 24, 36 and 48
ICECAP-A	Randomisation	✓ Week 12	✓ Weeks 24, 36 and 48
OxCAP-MH	Randomisation	✓ Week 12	✓ Weeks 24, 36 and 48
HEQ	Randomisation	✓ Week 12	✓ Weeks 24, 36 and 48

10.5 Run-in Phase

Participants who successfully complete the Screening Visit will enter into the run-in phase of the trial. The minimum duration of this phase will be 1 week. Eligibility for randomisation will usually be assessed after 2 weeks but the run-in phase can be extended, for example, if antidepressant treatment has been changed. If randomisation is delayed beyond 35 days after the screening visit the participant should be withdrawn from the trial and, if appropriate, re-screened at a future date when baseline assessments will be repeated.

The run-in phase is designed to minimise post-randomisation dropout by allowing assessment of symptom stability and of the likelihood that a participant will adhere to remotely recorded self-reports prior to randomisation.

10.6 Randomisation

10.6.1 Randomisation Visit

Participants may be randomised if they successfully complete the run-in phase (satisfy the additional inclusion criteria and no exclusion criteria apply, see sections 9.2 and 9.3). At the randomisation visit:

- Responses to study assessments will be reviewed, including self-reports on True Colours and the semi-structured interviews administered by the Research Assistants (RAs)

- For WOCBP (regardless of current use of contraception), a urine pregnancy test will be done. If a female participant does not meet the definition of a WOCBP, and a pregnancy test is not required, the reason should be documented in their medical notes
- For eligible participants, a blood sample will be taken to measure hs-CRP and, if optional consent has been given, an additional sample to be stored for future research and used for DNA extraction
- Clinician rating of depressive symptoms (QIDS-C) will be completed
- Measurement of blood pressure and pulse
- Identification of any adverse events
- Participants will also be asked to complete the decision-making task (see Appendix C)

10.6.2 Method of Randomisation

Randomisation will be performed centrally via an online randomisation system. The randomisation schedule will be designed by the trial statistician. Each participant will be randomised at a ratio of 1:1 to either pramipexole or a matched placebo. A non-deterministic algorithm will be used to produce treatment groups balanced for important prognostic factors by minimising separately on four variables:

- Trial Site
- Age (18-50, vs. >50)
- Gender (M/F)
- Baseline QIDS-SR_{16} severity (11-15 vs 16-20 vs >20)

The first 10 participants will be allocated treatment randomly without minimisation to avoid predictability. Subsequently, the minimisation algorithm will be applied with an allocation ratio that is not fully deterministic: there will be an 80% bias in favour of allocations that minimise the imbalance. Central randomisation will aid allocation concealment (Schulz, 1995).

As randomisation in this study is not time sensitive, a back-up randomisation system is not needed. If required, randomisations will be delayed until the online randomisation system is available again.

10.7 Blinding and code-breaking

Participants, investigators and researchers involved in trial recruitment and assessment visits will be blind to allocation. The pharmacy team dispensing trial medication will not be blinded. Participants wishing to remain on pramipexole treatment after the end of the treatment period may be unblinded at the discretion of the investigator where this is necessary to guide their ongoing treatment.

Participants will not be routinely unblinded at the end of the trial. Requests for unblinding may be made to the TMG in writing once the database has been locked for analysis. There are two exceptions:

- Emergency unblinding (see section 10.7.1)
- Unblinding for participants wishing to continue pramipexole treatment after the end of the trial treatment period (see section 10.7.2)

10.7.1 Emergency Unblinding

A phone number available 24 hours a day, 7 days per week will be provided to clinicians treating a participant experiencing a medical emergency. If required the psychiatrist will be able to access and reveal that participant's allocation. Participants will be provided with a card showing the number for clinicians to call to access this service. It is envisaged, however, that treatment of most medical emergencies will not

be affected by knowledge of a participant's allocation and that, therefore, unblinding will rarely be required.

10.7.2 Unblinding prior to study data lock

Participants who wish to continue taking pramipexole after the end of the trial treatment period (see section 11.1.8) are required to be unblinded prior to study data lock. The following principles ensure, as far as is possible, that the data is not changed or influenced following unblinding of these participants:

- Unless an emergency, unblinding will take place after the week 48 assessments have been completed
- Primary outcome data is recorded through True Colours (see section 10.4) and cannot be edited after questionnaire submission. Similarly, secondary outcome data (including health economics data and the cognitive task described in Appendix C) reported through True Colours cannot be edited after submission
- Safety and acceptability data is partially participant reported through True Colours. The non-participant reported data includes pulse and blood pressure data, and reported dose taken. These data are collected for up to 48 weeks and cannot practically be frozen prior to unblinding due to the short timeframe involved. However these are objective measures that would be unaffected by unblinding. It is also accepted that it will not be possible to freeze all adverse event data prior to unblinding due to the short time frame involved. SAEs will be reconciled on an ongoing basis from the start of the study
- Any changes to data in the study database (for example, to correct errors in data entry or to resolve data queries) will be fully documented and justified with an audit trail

10.7.3 Effectiveness of Blinding

For some participants, the escalation of pramipexole dose up to 2.5mg/day will be curtailed by the development of adverse events (at which point the dose will be reduced to the last tolerated). There is the potential using this regime that the pramipexole group will report more adverse events and, on average, take fewer tablets than the placebo group. However, a previous study in depressed patients (Cusin, 2013) reported no significant difference in adverse events between patients randomised to pramipexole (mean dose 1.35mg/day) and those to placebo. The original dose finding work for pramipexole in Parkinson's Disease (Richard, 1997) reported no difference in the incidence of adverse events for groups of patients on 1.5, 3 or 4.5mg of Pramipexole per day. It is not clear, therefore, to what extent the incidence of adverse events reported by the two groups will differ and to what extent there will be sufficient unblinding to introduce significant performance and ascertainment biases. The DMC will review adverse event data and the number of requests for unblinding at intervals during the study and will make recommendations to the TSC if required. Participants who have completed the week 48 assessments, or discontinue pramipexole earlier, will be asked whether they think they have been receiving active or placebo treatment. Any concerns around allocation concealment will be reviewed by the Trial Management Group.

10.8 Subsequent Trial Visits

The randomised phase will last for 48 weeks during which participants will be asked to attend four follow-up visits and to continue online self-reports using True Colours. Participants will also be contacted regularly by a trained RA, the schedule for RA contact is described in section 10.8.2.

10.8.1 Treatment Phase Visits

Au-Yeung SK, et al. Evid Based Ment Health 2022; 25:77–83. doi: 10.1136/ebmental-2021-300282

In addition to screening and randomisation visits, participants will be asked to attend clinic visits at Weeks 2, 6, 12 and 48-weeks post-randomisation.

These visits will include:

- verbal re-affirming of consent
- completion of QIDS-C
- review of online self-reports
- check for adverse events
- measurement of blood pressure and pulse
- check for changes in concomitant medication
- adjustment to pramipexole dose if required

In addition to this, at the Week 2 and Week 12 visits, participants will be asked to complete the decision making task (see Appendix C).

At the Week 48 visit, WOCBP will be asked to undergo a urine pregnancy test to confirm absence of pregnancy.

10.8.2 Research Assistant Contact Between Clinic Visits

From entry into the run-in phase until the collection of the primary outcome (week 12), participants will be contacted weekly by a trained trial RA. Following this, unless there is any cause for concern, the frequency of routine contact will reduce to 4-weekly until week 48. Contact will usually be by phone but, if this is not possible, an email will be sent. The treating clinician will be alerted if the RA is not able to make contact with the participant.

The RA will use a semi-structured interview plan to ask about adverse events including any increase in impulsive behaviour or suicidality, any changes in medication and any problems with adherence to self-reports. The QUIP-RS will be completed up to and including Week 12 and then 4-weekly throughout the randomised phase. The RA will also complete the scale during other contacts if they or the participant have concerns about impulse control. Participants will be asked about adverse events during each RA contact and the TSQM will be completed at weeks 1, 6, 12, 24 and 48.

The QUIP-RS and TSQM will be administered during the semi-structured interview as it would be important to pick up nuances in the response that may indicate there is a problem requiring escalation to a psychiatrist, which might not be identified if the collection was through online self-reports via True Colours.

Summary of RA contact between trial visits			
	Initiation	Weeks 0 – 12	Weeks 13 - 48
Interview (AEs suicidality, medication changes)	Screening	✓ Weekly	✓ 4-weekly
QUIP-RS	Screening	✓ Weekly	✓ 4-weekly
TSQM	Week 1 (after commencing trial medication)	✓ Weeks 1, 6, 12, 24 and 48	

The RA will report any concerns including an increase in the QIDS-SR$_{16}$ score for suicidal ideation and/or increase in impulsivity and reportable adverse events to the treating psychiatrist who will decide whether

any action is required. RAs will also monitor completion of online ratings regularly and contact participants who are not adhering to the schedule.

10.9 Sample Handling

10.9.1 Sample Handling for Standard of Care

A blood sample will be taken at the screening appointment for measurement of serum creatinine and calculation of eGFR and any other pre-treatment tests that the clinician thinks are indicated. The sample(s) will be sent to the local pathology laboratory for routine assay.

10.9.2 Sample Handling for Trial Purposes

CRP is an inflammatory marker that may predict response to specific antidepressants. A 7ml blood sample will be taken at the randomisation visit for measurement of hs-CRP. The sample will be sent to the PAX-D Coordinating Centre to be processed and frozen. Samples will be analysed in batches and may be kept for up to 1 year after the end of the trial. The results of this test will only be sent to the trial clinician if the result indicates a cause for concern.

10.9.3 Sample Handling for Future Research

If additional (optional) consent has been given, an additional 4ml blood sample will be taken at the randomisation visit and retained by the PAX-D Coordinating Centre for use in future research and for DNA extraction.

10.10 Early Discontinuation/Withdrawal of Participants

During the course of the trial a participant may choose to withdraw early from the trial treatment at any time. This may happen for a number of reasons, including but not limited to:

- The occurrence of what the participant perceives as an intolerable adverse event (AE)
- Inability to comply with trial procedures
- Participant decision

Participants may choose to stop treatment and/or study assessments but may remain on study follow-up. Participants may also withdraw from the study completely.

In addition, the Investigator may discontinue a participant from the trial treatment at any time if the Investigator considers it necessary for any reason including, but not limited to:

- Pregnancy
- Ineligibility (either arising during the trial or retrospectively having been overlooked at screening)
- Significant protocol deviation
- Significant non-compliance with treatment regimen
- An adverse event which requires discontinuation of the trial medication or results in inability to continue to comply with trial procedures
- Disease progression which requires discontinuation of the trial medication or results in inability to continue to comply with trial procedures
- Withdrawal of Consent

Au-Yeung SK, et al. Evid Based Ment Health 2022; 25:77–83. doi: 10.1136/ebmental-2021-300282

- Loss to follow-up

Participants who are judged by their treating psychiatrist to have temporarily lost capacity will discontinue study procedures and be allowed to resume the trial when capacity is regained.

Participants who stop trial treatment but do not withdraw consent to participation in the trial will continue with trial assessments and follow-up. For participants who withdraw consent, all trial data collection will be stopped. Data and samples collected prior to that withdrawal will be retained and the reason for withdrawal, if given, will be recorded in the trial Case Report Form (CRF). Participants discontinuing pramipexole or matched placebo will be advised on ongoing treatment and how to taper their dose of pramipexole or matched placebo safely (for further information see section 11.1.4).

The type of withdrawal and reason for withdrawal will be recorded in the CRF. If the participant is withdrawn due to an adverse event, the Investigator will arrange for follow-up until the adverse event has stabilised. If a participant is withdrawn from treatment due to pregnancy, consent will be sought for the pregnancy to be followed up to outcome.

10.11 Definition of End of Trial

The end of trial is set as the date of resolution of the last data query.

11 TRIAL INTERVENTIONS

11.1 Investigational Medicinal Product(s) (IMP) Description

PAX-D will compare pramipexole with a matched placebo. ModePharma, an MHRA-licensed wholesale distributor of human medicinal products, have been contracted to project manage and coordinate the supply of IMP for the trial. Development and manufacture of the matched placebo tablets will be undertaken by Custom Pharmaceuticals Limited (UK MIA(IMP) 4102). Clinical trials re-packaging, Annex-13 labelling and final QP release for clinical trial use will be undertaken by Wasdell Packaging Limited (UK MIA(IMP) 1411).

PRAMIPEXOLE	
Status of IMP	Pramipexole has an EU marketing authorisation for the treatment of Parkinson's Disease and Restless Leg Syndrome. It is not licensed for use in TRD.
Dosage and treatment duration	Treatment will commence following the randomisation visit and continue for up to 48 weeks. Participants will titrate toward a target dose of 2.5mg/day. The titration and tapering schedules are described in section 11.1.4.
Comparator	Matched placebo (see section 11.1.1)
Dosage form, packaging and labelling	Trial medication will be provided as:<br>• Pramipexole 0.18mg tablets (each tablet contains 0.25mg of pramipexole dihydrochloride monohydrate equivalent to 0.18 mg pramipexole)

	• Pramipexole 0.7mg tablets (each tablet contains 1mg of pramipexole dihydrochloride monohydrate equivalent to 0.7mg pramipexole)<br><br>The tablets will be bottled into induction sealed HDPE bottles (56 tablets per bottle) containing cotton with polypropylene child-resistant caps.<br><br>Labelling will meet requirements described in Volume 4. Good Manufacturing Practices, Annex-13. Manufacture of investigational medicinal products, July 2010.
Supply	Pramipexole or a matched placebo will be supplied free of charge and dispensed centrally to trial participants from the Oxford Health NHS Foundation Trust Clinical Pharmacy Support Unit (CPSU).
Packaging and storage conditions	The IMP should be stored in its original container.

11.1.1 Blinding of IMPs
Active and placebo will be packaged and labelled identically. Participants, investigators and the trial team will remain blind to allocation. As some participants may not tolerate the target dose of 2.5 mg/day the pharmacy will be unblinded to allow for appropriate quantities of IMP to be dispensed according to the prescription. The bottle labels will include a tear-off section indicating the contents (active or placebo) which will be removed in the pharmacy at the point of dispensing to render the bottle blind.

11.1.2 Storage of IMP
Following manufacture the IMP will be stored by Wasdell Packaging Limited, and distributed in batches to the Oxford Health NHS Foundation Trust Clinical Pharmacy Support Unit (CPSU) where it will be kept until dispensed to trial participants.

11.1.3 Provision of trial medication to participants
Prescribers will be medically qualified doctors delegated the responsibility for prescription of trial medication. Packs of trial medication will be posted to participants' nominated address* (which will usually be their home address) from the Oxford Health NHS Foundation Trust CPSU. Posting medication was found in other trials (e.g. BALANCE – Geddes et al., 2009; CEQUEL – Geddes et al., 2016) to be a safe and effective way to provide medication to participants between trial visits.

Completed trial prescriptions should be sent securely to the CPSU dispensary inbox

dispensary@oxfordhealth.nhs.uk

Participants will be asked to confirm receipt of trial medication by phone or email. If confirmation is not received within two days of posting, a member of the trial team will contact the participant. If they are

unable to make contact, the participant's clinician will be informed. If the participant cannot be contacted further packs of trial medication will not be dispensed.

Packs will include a written schedule indicating how many tablets should be taken each day. The dosing schedule will also be explained to patients during the randomisation visit. If subsequent dose changes need to be made the participant will be provided with a revised schedule.

* Where the nominated address is a pharmacy or GP surgery the trial team will confirm that this is acceptable before medication is mailed and will clarify that the request is to store the pack containing medication to be handed to the participant unopened. An RA will confirm receipt and, if the pack has not been collected, contact the participant and follow up as above.

11.1.4 Titration and tapering of pramipexole/placebo

For most participants, initiation of pramipexole treatment will be according to a 4-week titration schedule starting at 0.25mg/day in a single dose at night for 3 days. Thereafter the dose will be increased by 0.25mg/day every 3 days. The target dose will be 2.5mg/day but titration will be based on tolerability and response. The titration schedule may be amended at the discretion of the treating psychiatrist (e.g. patients may prefer to take their pramipexole during the day, and this is acceptable).

**Titration Schedule for Pramipexole**	
Days	Dose mg/day
1 - 3	0.25
4 - 6	0.5
7 - 9	0.75
10 -12	1.0
13 - 15	1.25
16 - 18	1.5
19 - 21	1.75
22 – 24	2.0
25 – 27	2.25
28 –	2.5

Participants who are unable to tolerate an increased dose of pramipexole will be advised to reduce the dose to the highest tolerated. Participants will remain on this dose throughout the remainder of the trial. No re-titration will be attempted.

For participants discontinuing pramipexole or matched placebo, as for titration, dose reductions during tapering will be made every 3 days. This will reduce the risk of developing dopamine withdrawal syndrome, which may be caused by abrupt withdrawal of dopaminergic therapy.

**Tapering Schedule for Pramipexole**	
Days	Dose mg/day
1 - 3	2.0
4 - 6	1.25
7 - 9	0.75
10 -12	0.5
13 - 15	0.25
Medication stopped	

11.1.5 Compliance with Trial Treatment

Participants will be asked to report adherence to trial treatment and number of tablets remaining at each RA contact. Returned tablet counts will be used to provide a further test of adherence. The RA will inform the treating psychiatrist if patients are missing >10% of their pramipexole doses.

11.1.6 Accountability of the Trial Treatment

All medication will be accounted for by CPSU and allocated medication will be recorded on a Drug Allocation Log for each participant. Participants will be provided with freepost return envelopes to enable them to return unwanted medication to the pharmacy free of charge.

11.1.7 Concomitant Medication

Participants will be asked to avoid any changes in any concomitant medication during the first 12 weeks of the randomised phase unless clinically mandatory.

Participants reporting nausea after commencing pramipexole treatment (see section 11.4.1) may be prescribed an antiemetic. Dopamine antagonists that cross the blood-brain barrier (e.g. metoclopramide) should be avoided. As guidance, domperidone is suggested as an appropriate antiemetic.

People taking antipsychotic medication and any other medications known to interfere with pramipexole metabolism (including cimetidine, memantine and methyldopa) will be excluded from the trial. If these are commenced out of clinical necessity participants will discontinue pramipexole. People who have taken pramipexole for 2 weeks or more in the past will also be excluded from the trial.

11.1.8 Post-trial Treatment

When participants reach 48-weeks post-randomisation they will attend a final trial visit when they and the investigator will decide on ongoing treatment options. For participants stopping pramipexole medication a tapering schedule will be provided to avoid withdrawal effects. As pramipexole is not licensed for use in TRD, availability outside of the study varies across the UK and would be according to the local Trust prescribing policy. For participants wanting to continue pramipexole, local provision would need to be arranged.

11.2 Other Treatments (non-IMPS)

Pharmacological treatment of TRD usually involves antidepressant medication. In PAX-D trial pramipexole will be added to an antidepressant that the participant is prescribed outside of the trial.

11.3 Other Interventions

New courses of psychological therapy should not be commenced in first 12 weeks of trial.

11.4 Tolerability and safety

11.4.1 Side-effects of pramipexole

Participants will be provided with information about the adverse effects of pramipexole and given advice on when to seek medical attention. Common adverse effects include nausea, sedation and dyskinesia (unusual, involuntary movements). More serious effects include somnolence, sudden sleep onset and impulse control disorders.

Tolerability of the IMP will be reviewed during the trial visits at Weeks 2, 6 and 12, and during RA contacts throughout the trial (see section 10.8.2). In addition, participants will be provided with the contact number for their Trial Site and asked to make contact should they develop any adverse events.

11.4.2 Impulse control symptoms

Pramipexole, in common with other dopaminergic drugs, can induce impulse control symptoms (e.g. increased gambling, self-initiated escalation of dosage) in a sub-group of patients. The risks associated with this adverse effect will be mitigated in 4 key ways:

- A history of impulse control problems is a strong predictor of the development of such symptoms with dopaminergic drugs). This is therefore an exclusion criterion and the PIS will clearly describe the potential for the medication to induce impulse control difficulties and state that potential participants who have experienced these problems in the past should not enrol in the trial
- At screening, symptoms suggestive of previous impulse control disorders (including harmful alcohol or drug use (prescription and illegal), gambling or sexual behaviours) will be assessed and potential participants with such symptoms will not be enrolled
- During the trial, the RAs will regularly contact participants (see section 10.8.2). This contact will include completion of the QUIP-RS. This questionnaire is specifically designed to test for the presence of symptoms of impulse control difficulties including increase in medication use. Participants showing an increase in this score (of 2 or more points from baseline) will trigger a clinical assessment by the local trial team. Where necessary these trial assessments will be supplemented by additional visits as deemed necessary by the treating clinical team
- Difficulties with impulse control can lead to excessive use of the trial medication. The semi-structured telephone calls to be made by RAs will include questions designed to elicit overuse of medication and will include tablet counts. In addition to this, any requests from participants for packs of medication before new packs are due will be referred to a trial psychiatrist or the local clinical team. Participants who are identified as inappropriately escalating the dose of the medication will (as for other impulse control disorders) be referred to the local clinical team for management of a monitored dose reduction.

11.4.3 Management of Overdose

There is no clinical experience with massive overdose and general supportive measures are recommended, along with gastric lavage, intravenous fluids, administration of activated charcoal and electrocardiogram monitoring. If signs of central nervous system stimulation are present, a neuroleptic agent may be indicated.

12 SAFETY REPORTING

12.1 Adverse Event Definitions

Date and version No: Version 3, 24/08/2021

Adverse Event (AE)	Any untoward medical occurrence in a participant to whom a medicinal product has been administered, including occurrences which are not necessarily caused by or related to that product.
Adverse Reaction (AR)	An untoward and unintended response in a participant to an investigational medicinal product which is related to any dose administered to that participant.<br>The phrase "response to an investigational medicinal product" means that a causal relationship between a trial medication and an AE is at least a reasonable possibility, i.e. the relationship cannot be ruled out.<br>All cases judged by either the reporting medically qualified professional or the Sponsor as having a reasonable suspected causal relationship to the trial medication qualify as adverse reactions.
Adverse Event of Special Interest (AESI)	An AE that is of scientific and medical concern to the Trial Management Group for which rapid communication is required. The AESI may not meet the standard criteria for seriousness and it may occur outside the standard AE reporting timeframes for the trial. The AE(s) of special interest for this trial are listed in section 12.6.
Serious Adverse Event (SAE)	A serious adverse event is any untoward medical occurrence that:<br>• results in death<br>• is life-threatening<br>• requires inpatient hospitalisation or prolongation of existing hospitalisation<br>• results in persistent or significant disability/incapacity<br>• consists of a congenital anomaly or birth defect*.<br>Other ‘important medical events’ may also be considered a serious adverse event when, based upon appropriate medical judgement, the event may jeopardise the participant and may require medical or surgical intervention to prevent one of the outcomes listed above.<br>NOTE: The term "life-threatening" in the definition of "serious" refers to an event in which the participant was at risk of death at the time of the event; it does not refer to an event which hypothetically might have caused death if it were more severe.<br>*NOTE: Pregnancy is not, in itself an SAE. In the event that a participant or his/her partner becomes pregnant whilst taking part in a clinical trial or during a stage where the foetus could have been exposed to the medicinal product (in the case of the active substance or one of its metabolites having a long half-life) the pregnancy should be followed up by the investigator until delivery for congenital abnormality or birth defect, at which point it would fall within the definition of “serious”.

Au-Yeung SK, *et al. Evid Based Ment Health* 2022; 25:77–83. doi: 10.1136/ebmental-2021-300282

Serious Adverse Reaction (SAR)	An adverse event that is both serious and, in the opinion of the reporting Investigator, believed with reasonable probability to be due to one of the trial treatments, based on the information provided.
Suspected Unexpected Serious Adverse Reaction (SUSAR)	A serious adverse reaction, the nature and severity of which is not consistent with the Reference Safety Information for the medicinal product in question set out:<br>• in the case of a product with a marketing authorisation, in the approved summary of product characteristics (SmPC) for that product<br>• in the case of any other investigational medicinal product, in the approved investigator's brochure (IB) relating to the trial in question.

NB: to avoid confusion or misunderstanding of the difference between the terms "serious" and "severe", the following note of clarification is provided: "Severe" is often used to describe intensity of a specific event, which may be of relatively minor medical significance. "Seriousness" is the regulatory definition supplied above.

12.2 Assessment results outside of normal parameters as AEs and SAEs

The trial includes minimal laboratory tests. At screening a blood sample is taken for serum creatinine and eGFR to confirm eligibility; abnormalities in these results do not require reporting as AEs or SAEs because they are outside the safety reporting period (see section 12.7). A blood sample for hs-CRP is taken at randomisation and analysed centrally. Abnormalities in this result will be logged as AEs or SAEs, and clinically significant results will be notified to the recruiting clinician.

12.3 Assessment of Causality

The relationship of each adverse event to the trial medication must be determined by a medically qualified individual according to the following definitions:

Related: The adverse event follows a reasonable temporal sequence from trial medication administration. It cannot reasonably be attributed to any other cause.

Not Related: The adverse event is probably produced by the participant's clinical state or by other modes of therapy administered to the participant.

12.4 Contraception and Pregnancy

The effect of pramipexole on pregnancy has not been investigated in humans but the advice in the Summaries of Product Characteristics (SmPC) is that it should not be used during pregnancy unless clearly necessary. Pregnancy, lack of agreement by women of childbearing potential to use effective contraception and breast-feeding are all exclusion criteria.

It is a requirement that WOCBP use effective contraception during the at-risk period, considered to be from the randomisation visit until two weeks after pramipexole has been stopped. The following methods of contraception are acceptable:

- Combined oestrogen and progestogen contraception (oral, injectable, or implantable)
- Progestogen-only hormonal contraception (oral, injectable, or implantable)
- Intrauterine device
- Intrauterine hormone-releasing system
- Male or female condom with or without spermicide
- Cap, diaphragm or sponge with spermicide
- Bilateral tubal occlusion
- Vasectomised partner
- Sexual abstinence (only if defined as refraining from heterosexual intercourse during the at risk period). See note below.

Note: Sexual abstinence is acceptable only as true abstinence: when this is in line with the preferred and usual lifestyle of the patient. Periodic abstinence (e.g., calendar, ovulation, symptothermal, post-ovulation methods) and withdrawal are not acceptable methods of contraception.

In the event of pregnancy, advice on tapering pramipexole will be given and alternative treatment provided if indicated. Participants who become pregnant will be asked to consent to be followed up until the end of the pregnancy and any complications or other adverse events experienced by mother and/or baby classified as in 10.1 and reported as required.

There is no information to indicate that there are any risks associated with conception when the male is taking pramipexole and therefore follow-up of pregnancies of female partners of male participants will not be carried out.

12.5 Procedures for Reporting Adverse Events

All AEs observed by the Investigator or reported by the participant that occur at any point from the participant signing the informed consent form until 2 weeks after pramipexole has been stopped, will be recorded on the Adverse Event Log, whether or not attributed to trial medication.

AEs considered related to the trial medication as judged by a medically qualified investigator or the Sponsor, will be followed until resolution or until the event is considered stable. Follow-up information should be provided as necessary.

It will be left to the Investigator's clinical judgment to decide whether or not an AE is of sufficient severity to require the participant's removal from treatment. A participant may also voluntarily withdraw from treatment due to what they perceive as an intolerable AE (see section 10.10).

12.6 Adverse Events of Special Interest (AESI)

The following adverse events of special interest for pramipexole must be collected from the participant signing the informed consent form until two weeks after the last dose of pramipexole:

- Impulse control disorder

Au-Yeung SK, *et al. Evid Based Ment Health* 2022; 25:77–83. doi: 10.1136/ebmental-2021-300282

- Psychosis (including psychotic depression)

They must be reported on an SAE form regardless of their seriousness within 24 hours of becoming aware of the event. AESIs must be followed up until resolution. In terms of causality and expectedness, symptoms of impulse control disorders and psychosis would generally be considered expected events for dopamine agonists such as pramipexole, and are listed in the reference safety information. AESIs will be listed within the DSUR (see section 12.10).

12.7 Reporting Procedures for Serious Adverse Events

SAEs occurring before the date the participant starts taking trial medication will be reported on the study database.

All SAEs occurring from the date the participant starts taking trial medication until two weeks after the last dose of pramipexole, other than those defined in this protocol as not requiring reporting must be reported on the SAE Reporting Form to the Sponsor or delegate immediately or within 24 hours of the site study team becoming aware of the event being defined as serious.

SAEs will also be reviewed at regular intervals by the TSC and DMC.

Date and version No: Version 3, 24/08/2021

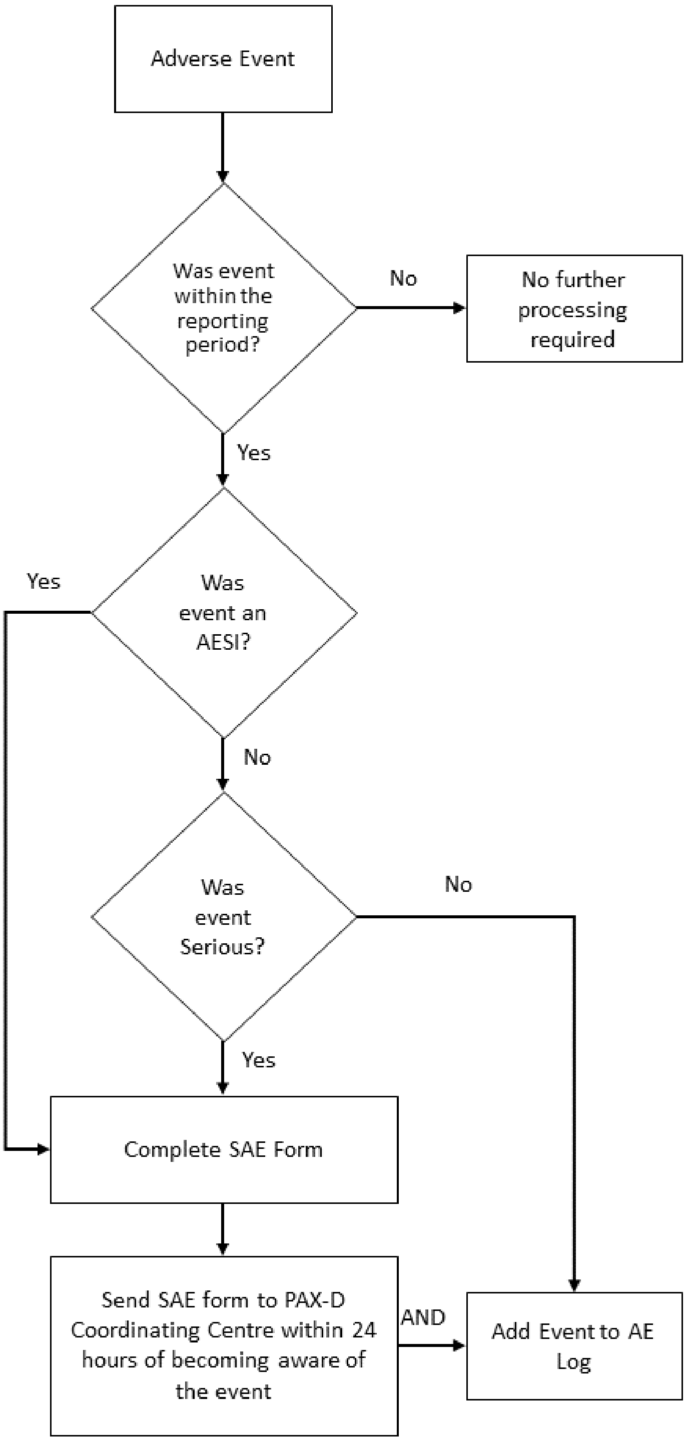

Au-Yeung SK, *et al. Evid Based Ment Health* 2022; 25:77–83. doi: 10.1136/ebmental-2021-300282

12.7.1 Events exempt from immediate reporting as SAEs

Hospitalisation for a pre-existing condition, including an elective procedure planned prior to study entry, which has not worsened, does not constitute a serious adverse event.

12.7.2 Procedure for immediate reporting of Serious Adverse Events

All SAEs other than those defined in the protocol as not requiring reporting must be reported on the SAE reporting form to the PAX-D Coordinating Centre within 24 hours of the local team becoming aware of the event. All SAE information must be recorded on an SAE form and scanned and emailed or faxed, to the PAX-D Coordinating Centre using the details printed on the SAE form. Additional and further requested information (follow-up or corrections to the original case) will be detailed by signed and dated corrections to the original form and sent to the coordinating centre as detailed above. SAEs that are reported late must be accompanied by an explanation for this. Coordinating centre staff will perform an initial check of the report and request any additional information from the local team. The coordinating centre will ensure the SAE is sent within 1 business day of receipt for review by the Chief Investigator (CI) or a safety delegate.

12.8 Expectedness

Expectedness will be determined by the CI, or their delegate, according to the approved RSI (Summary of Product Characteristics for Pramipexole).

12.9 SUSAR Reporting

All SUSARs will be reported by the sponsor delegate to the relevant Competent Authority and to the REC and other parties as applicable. For fatal and life-threatening SUSARS, this will be done no later than 7 calendar days after the Sponsor or delegate is first aware of the reaction. Any additional relevant information will be reported within 8 calendar days of the initial report. All other SUSARs will be reported within 15 calendar days. Treatment codes will be unblinded for specific participants.

Principal Investigators will be informed of all SUSARs for the relevant IMP for all studies with the same Sponsor, whether or not the event occurred in the current trial.

12.10 Development Safety Update Reports

The CI will submit (in addition to the expedited reporting above) DSURs once a year throughout the clinical trial, or on request, to the Competent Authority (MHRA in the UK), Ethics Committee, HRA (where required), and Sponsor.

13 STATISTICS

13.1 Statistical Analysis Plan (SAP)

The statistical aspects of the study are summarised here, details will be fully described in a statistical analysis plan (SAP).

Au-Yeung SK, *et al. Evid Based Ment Health* 2022; 25:77–83. doi: 10.1136/ebmental-2021-300282

13.2 Description of Statistical Methods

The primary outcome (change in QIDS-SR_{16} between baseline and week 12) will be analysed using a generalised linear mixed model utilising data collected at all time points from randomisation and including the baseline outcome as a fixed effect.

Continuous secondary outcomes will be analysed using generalised linear models.

The dichotomous secondary outcomes will be analysed using a logistic mixed effects regression model. These analyses will include a fixed effects randomised group and baseline level of the QIDS-SR_{16}, with participants and trial site accounted for as random effects. Minimisation variables will be included as explanatory factors in the models. Mediational analysis will test whether changes in reward sensitivity mediate the effect of pramipexole on depressive symptoms. Given the sample size proposed for this trial, the mediational analysis will provide >80% power to detect a mediation effect if both the effect size of pramipexole on reward sensitivity and the of reward sensitivity on symptoms of depression are greater than 0.26 (Fritz & MacKinnon, 2007).

Health economic data analysis will assess group differences in quality of life, wellbeing and work performance using a cost-utility analysis described in section 13.7.

13.3 Sample Size Determination

Considering a 3 point difference on QIDS-SR_{16} score between pramipexole and placebo at 12 weeks to be clinically important with standard deviation of the scores of 5.4 (based on those observed in the CEQUEL trial: Geddes et al., 2016), produces a standardised effect size of 0.56. The sample size to test differences between groups at 90% power and at a type one error rate of 5% would be 68 per group (136 total), increasing with 20% drop-out to 170 total. To test a similar sized difference in reward sensitivity and to ensure that type one error for both the depression score and the reward sensitivity tests is 0.05 or below, the sample size calculation can be determined using an alpha of 0.025. At an alpha of 0.025 and power of 90%, the required sample size would be 81 per group. Allowing for a 20% loss to follow-up would then increase the sample size to 102 per group (204 total).

13.4 Analysis Populations

The primary and efficacy-based secondary analyses will be performed using an intention-to-treat approach for all randomised participants. Analyses of the mechanistic secondary outcomes and health economic outcomes will be performed in the set of subjects who have the data required for the specific analyses (i.e. no imputation will be performed for these analyses). Acceptability analyses will be performed on a subgroup of participants and trial clinicians who provide separate consent for that aspect of the trial.

13.5 Stopping Rules

Stopping rules will be agreed by the TSC and DMC at their first meeting, and documented in the appropriate committee charters. It is expected that recruitment will be assessed 12 months after multi-centre recruitment commences to determine if a minimum recruitment target is being achieved to deliver a successful trial. The decision to terminate the trial will be made by the TSC.

Au-Yeung SK, *et al. Evid Based Ment Health* 2022; 25:77–83. doi: 10.1136/ebmental-2021-300282

13.6 Procedure for Accounting for Missing, Unused, and Spurious Data

Missing values in all outcomes will be inspected and reported across treatment group and follow-up time. For variables measured at baseline and one follow-up interval, logistic regression will be performed to explore the effects of treatment and baseline measure on 'missingness' of each outcome. For outcome variables repeated and measured for the full 48 weeks of the follow-up period, multilevel logistic regression will be performed to examine the influence of treatment status, baseline measures and country on missingness of each outcome. The missing value pattern(s) and logistic regression results will be used to inform missing value imputation under the 'Missing At Random' assumption.

13.7 Health Economics Analysis

The main health economic analysis will include: (i) a detailed patient-level cost analysis of health, social care and other broader societal costs for both the pramipexole and placebo arms of the trial and (ii) an incremental within-trial economic evaluation comparing the pramipexole and placebo arms of the trial in terms of their costs and outcomes over the 48 week trial follow-up period.

The cost analysis will be based on resource use data collected as part of a bespoke Health Economics Questionnaire (HEQ) whose development was based on previous versions of the Client Service Receipt Inventory (CSRI) instrument (Chisholm et al., 2000, Patel et al., 2013), a widely-used and validated instrument for collection of resource use data in mental health. Collected data will include all hospital and community health and social services, medication, productivity losses, informal care and patient's travel expenses. Costing will be conducted using national-level unit costs from the UK for a common year, e.g. PSSRU Unit Costs Database (Curtis & Burns, 2017) and BNF (Joint Formulary Committee, 2018). Lost productivity costs due to absenteeism or presenteeism will be estimated using the human capital approach where time off work is multiplied by the average daily national salary for participants who are employed or self-employed (NICE 2009b; Tarricone, 2006).

The primary health economic analysis will be a cost-utility analysis from a health and social care perspective where quality-adjusted life years (QALYs) will be calculated using utility values from the EQ-5D-5L health-related quality of life questionnaire as recommended by most health technology assessment agencies (Drummond et al., 1997; EuroQoL Group, 199;, NICE, 2013). Health states will be valued by using the common tariff set from the UK and results will be expressed in an incremental cost-effectiveness ratio (ICER). Secondary economic analyses using the ICECAP-A (Al-Janabi et al., 2012) and the OxCAP-MH (Simon et al., 2013) capability indices as outcome measures will be also carried out. The capability states measured by the ICECAP-A will be valued by the tariff set for the UK (Flynn, 2015). A similar tariff set will be developed for the OxCAP-MH alongside this trial and will subsequently be applied in the valuation. Further analyses will estimate cost-effectiveness from a societal perspective.

All economic analyses will be on an intention-to-treat basis. Relevant outcome and resource use data will be collected electronically from participating patients at pre-defined time points throughout the trial. Data on HEQ, EQ-5D-5L, ICECAP-A and OxCAP-MH will be collected on five occasions through True Colours (see section 10.4).

Missing data will be reported and their potential impact on the results in contrast to the complete cases dataset will be investigated in a sensitivity analysis using multiple imputation based on the missing at random assumption (White et al., 2011; Little & Rubin, 2002). Further sensitivity analyses will address the potential impact of uncertainties in unit costs and outcomes.

Results will be reported as means with standard deviations or as mean differences with 95% confidence intervals. Differences in mean costs and effects will be compared in a regression framework with a p-value less than 5% considered as statistically significant. Non-parametric bootstrapping (Efron & Tibshirani, 1993) from the cost and effectiveness data will be used to generate a joint distribution of the mean incremental costs and effects for the options under comparison and to calculate the 95% confidence intervals of the incremental cost-effectiveness ratios (ICERs). Uncertainty around the main cost-effectiveness estimates will be represented by cost-effectiveness acceptability curves (CEACs) using the net benefit approach (Fenwick & Byford, 2005; Stinnett & Mullahy, 1998). CEACs show the probability that each option is cost-effective to a range of maximum values (ceiling ratio) that a decision maker might be willing to pay for an additional unit of improvement in outcomes.

13.8 Internal Pilot and Evaluation of Trial Performance

The internal pilot will be evaluated after 6 months of recruitment at one site. It will address 3 distinct trial components:

- Qualitative interviews will be conducted with local referring general practitioners and consultant psychiatrists from community treatment teams. Barriers to recruitment will be identified using a framework analysis designed for depression RCTs
- Qualitative interviews with participants, and of consultant psychiatrists from the treating team with responsibility for the participants will be undertaken, selected on the basis of reported difficulties or greater than anticipated drop-out in the pre-randomisation process
- The first 20 participants randomised to trial medication will be followed carefully during the active trial phase (i.e. to Week 12). Retention rates will be examined and reasons for discontinuation be explored. The proposed dose escalation strategy is based on published experience from the Fawcett (2016) study. However, this paper describes a large range of final pramipexole doses and a need to adapt treatment to individual patient response and adverse events. A key question which will be addressed is whether any adjustment of the dose escalation is needed and/or whether the titration schedule should be adjusted. Qualitative interviews of participants' experiences of having difficulty with continuing or discontinuing medication will be undertaken using a framework analysis based on experiences of escalating pramipexole for treatment resistant depression. In addition, data quality (average response times and accuracy) from the decision making task will be assessed to ensure that participants understand the task and are completing it appropriately

Following the end of the internal pilot and beginning of the multicentre phase trial performance will continue to be evaluated:

- All participants entering weeks 12 to 48 on treatment will be carefully evaluated until at least 10 have passed the 6 month point. The rate of participants remaining on trial medication will be measured and qualitative interviews of participants and their treating clinicians will take place to assess reasons for treatment discontinuation
- The internal pilot data from each phase of the trial will be presented to our Patient and Public Involvement (PPI) group for discussion and further insights (see section 18.9). The data and these insights will then be presented to the Trial Management Group together with comments from the Trial Manager, Trial Statistician, and Trial RAs. For each phase a decision will be reached as to whether any alterations to the trial protocol or procedures are required. These decisions will be presented to the DMC for consideration and recommendation to the TSC.

Unless substantial alteration to the trial protocol is required data from participants recruited into the internal pilot will be included in the analysis of the main trial.

Identified good practice in the trial will be disseminated across trial sites. Remedial steps which may be required include direct advertising to patients about the trial to increase recruitment and discussions with Clinical Research Networks (CRNs) around support for local PIs and adjustment of the dose escalation process. The ability to adjust a trial's protocol in this population of vulnerable patients with a severe illness has previously been shown to enable major trial completion (e.g. BALANCE – Geddes et al., 2009).

14 DATA MANAGEMENT

The data management aspects of the study are summarised here with details fully described in the Data Management Plan.

14.1 Source Data

Source documents are where data are first recorded, and from which participants' CRF data are obtained. These include, but are not limited to, hospital records (from which medical history and previous and concurrent medication may be summarised into the CRF), clinical and office charts, laboratory and pharmacy records, diaries, microfiches, radiographs, and correspondence.

In PAX-D data from True Colours self-reports will be classified as source data. CRF entries will be considered source data if the CRF is the site of the original recording (e.g. there is no other written or electronic record of data). This includes the informed consent form and any screening logs, and any CRFs and documentation completed by the RAs during the semi-structured interviews.

14.2 Use of Personal Data

Coordinating Centre staff will need access to participants' names and contact details to enable them to make the regular email/phone contacts required by the protocol. Personal data held at the Coordinating Centre will be stored securely and will only be accessible to staff who need to contact participants.

Staff at the Oxford Health Clinical Pharmacy Support Unit will also need this information in order to dispense and mail medication to participants.

14.3 Access to Data

Direct access to trial data, source data and medical records will be granted to authorised representatives from the Sponsor, host institution and the regulatory authorities to permit trial-related monitoring, audits and inspections.

14.4 Data Recording and Record Keeping

Data on the primary and secondary outcomes will be collected by participant's self-report using the True Colours system (see section 10.4). Text or electronic notification reminders for completion will be sent as appropriate, with the option for telephone completion in the event of a delayed response to ensure a high response rate.

Trial data will be collected using paper or electronic CRFs and transferred for storage in the clinical database. The individual participant data will be identified by a study participant specific number only. A separate administrative system will be used to store the participant's name and other identifying details. Clinical and administrative systems will be linked by the participant's study number only. All data will be processed in line with data management Standard Operating Procedures and Trial Specific Working Instructions. Access to the electronic data will be strictly controlled using individual passwords for all staff accessing the electronic databases.

Electronic files will be stored on a restricted access server held in a secure location. In line with University Department of Psychiatry policy, authorised access to the Department is via an electronic swipe card entry system and individual rooms are kept locked when not occupied. Authorised staff will process data via a secure network which requires individual login name and password. No data will be stored on individual workstations. The data is backed up automatically overnight to an offsite storage area accessed by authorised personnel via electronic tag and key-pad systems. All paper and electronic data will be stored securely in compliance with data protection regulations.

15 QUALITY ASSURANCE PROCEDURES

15.1 Risk assessment

The trial will undergo a risk assessment prior to starting, which will be reviewed at regular intervals to reflect significant changes to the protocol or outcomes of monitoring activities.

15.2 Monitoring

The PI will be responsible for the running of the trial at their site. This will include ensuring successful recruitment, staff education and training, and study data completeness and quality.

A monitoring plan will be developed based on the risk assessment and will include triggers for 'for cause' site monitoring. No other routine monitoring or auditing will be conducted unless central monitoring triggers cause to do so.

15.3 Trial committees

15.3.1 Trial Steering Committee

A Trial Steering Committee (TSC) will be convened to review the trial protocol, receive progress (including recruitment) and safety reports throughout the trial and assess the implications for the trial of any new research data that becomes available. In addition to a chair person, the committee will include independent and lay members and meetings will be attended by the CI and Trial Manager.

The TSC will meet before the start of the trial and will then meet to review the internal pilot (see section 13.8). Thereafter the committee will arrange a meeting at least annually. All SAEs will be reported periodically to the Committee Chair and SUSARS will be reported to the Committee Chair immediately. The Committee will hold meetings as required (which may be by teleconference) and make recommendations to the CI and/or Sponsor.

15.3.2 Data Monitoring Committee

A Data Monitoring Committee (DMC) will be convened to review unblinded comparative data and make recommendations to the TSC on whether there are any ethical or safety reasons why the trial should not continue. All SAEs will be reported periodically to the Committee Chair and SUSARS will be reported to the Committee Chair immediately. The DMC will review recruitment and the interim data from the internal pilot and convene at least annually thereafter. The DMC will be independent of the PIs and the TSC.

15.3.3 Trial Management Group

The trial will be supervised on a day-to-day basis by the Trial Management Group (TMG). This group reports to the TSC which is responsible to the trial sponsor. The Trial Management Group will meet regularly during the trial. The TMG will include the Chief Investigator, clinicians and experts from relevant specialties and trial staff from the University Department of Psychiatry.

The TMG will review substantial amendments to the protocol prior to submission to the REC and MHRA. All PIs will be kept informed of substantial amendments and are responsible for their prompt implementation.

16 PROTOCOL DEVIATIONS

A trial related deviation is a departure from the ethically approved trial protocol or other trial document or process (e.g. consent process or IMP administration) or from Good Clinical Practice (GCP) or any applicable regulatory requirements. Any deviations from the protocol will be documented in a protocol deviation form and filed in the trial master file.

17 SERIOUS BREACHES

The Medicines for Human Use (Clinical Trials) Regulations contain a requirement for the notification of "serious breaches" to the MHRA within 7 days of the Sponsor becoming aware of the breach.

A serious breach is defined as “A breach of GCP or the trial protocol which is likely to affect to a significant degree –

(a) the safety or physical or mental integrity of the subjects of the trial; or

(b) the scientific value of the trial”.

In the event that a serious breach is suspected the Sponsor must be contacted within 1 working day. In collaboration with the CI the serious breach will be reviewed by the Sponsor and, if appropriate, the Sponsor will report it to the REC committee, Regulatory authority and the relevant NHS host organisation within seven calendar days.

18 ETHICAL AND REGULATORY CONSIDERATIONS

18.1 Declaration of Helsinki

The Investigator will ensure that this trial is conducted in accordance with the principles of the Declaration of Helsinki.

18.2 Guidelines for Good Clinical Practice

The Investigator will ensure that this trial is conducted in accordance with relevant regulations and with the principles of Good Clinical Practice.

18.3 Approvals

Following Sponsor approval the protocol, informed consent form, participant information sheet, and any proposed advertising material will be submitted to an appropriate Research Ethics Committee (REC), HRA (where required), regulatory authorities (MHRA in the UK), and host institution(s) for written approval.

The Investigator will submit and, where necessary, obtain approval from the above parties for all substantial amendments to the original approved documents.

18.4 Other Ethical Considerations

Any person with a mental illness might, at times, be considered to be vulnerable. The design of the trial includes measures to identify particular concerns for trial participants. Suicidality will be assessed by the investigator at visit and the item relating to this in the QIDS-SR_{16} will be reviewed frequently.

RAs who contact participants will be fully trained and will be provided with a brief semi-structured interview plan. The RAs will have immediate access to a psychiatrist if required. If a participant were to become unwell during the trial permission would be sought to contact their care team (GP or psychiatrist). It will be made clear to all participants that taking part in the trial is in addition to routine care and that, if they feel unwell during the course of the trial, they should contact their GP or care team.

Participants will be asked to complete a number of online rating scales. Some (the QIDS-SR_{16}, ALTMAN and GAD-7) are used in routine practice and will therefore enhance patient care. The schedules for the remaining online scales have been kept to the minimum required to provide robust information on the effects of pramipexole and may also help participants to manage their depression.

Participants with TRD currently taking antidepressant treatment will have a 50% chance of this being augmented by placebo. Since there is uncertainty about the benefits of add-on treatments for participants with TRD and all participants will be taking a standard antidepressant, the addition of placebo rather than active medication is justified. Furthermore, participants' depressive symptoms will be monitored frequently and, if required, additional treatment will be prescribed.

18.5 Progress Reporting

The CI shall submit once a year throughout the clinical trial, or on request, an Annual Progress Report to the REC, HRA (where required), host organisation, funder (where required) and Sponsor. In addition, an End of Trial notification and final report will be submitted to the MHRA, the REC, host organisation and Sponsor.

18.6 Participant Confidentiality

The study will comply with the General Data Protection Regulation (GDPR) and Data Protection Act 2018, which require data to be de-identified as soon as it is practical to do so. The processing of the personal

data of participants will be minimised by making use of a unique participant study number only on all study documents and any electronic database(s), with the exception of the registration CRF, which will collect identifiers and contact details. All documents will be stored securely and only accessible by study staff and authorised personnel. The study staff will safeguard the privacy of participants' personal data.

18.7 Expenses and Benefits

All participants will be able to claim reasonable travel expenses, including costs of parking for research-related visits and, for lengthy visits, will be provided with refreshments. No incentives will be provided for participation in the trial.

18.8 Peer Review

The trial has undergone Peer review through the process of grant application and funding award by NIHR EME.

18.9 Patient and Public Involvement

A Patient Advisory Group (PAG) will be established to provide insight from those with lived experience of TRD. The group will be comprised of patients and carers, and its membership will be between 4 – 6 people.

The group will help ensure the research is both relevant and feasible, and its members will be involved in:

- Finalising trial design making sure what we are asking participants to do is reasonable
- Ensuring recruitment and participant information is presented in a way that is understandable
- Evaluation of the pilot phase
- Discussing the interpretation of the data
- Planning the dissemination of results

The Patient Advisory Group will be coordinated through the Oxford Health Biomedical Research Centre.

18.10 Conflicts of Interest

The PAX-D Investigators have no conflicts of interest to declare.

19 FINANCE AND INSURANCE

19.1 Funding

The trial is funded by the National Institute for Health Research (NIHR) Efficacy and Mechanism Evaluation (EME) programme (ref: 16/127/17). The views expressed are those of the author(s) and not necessarily those of the NHS, the NIHR, or the Department of Health.

19.2 Insurance

The University has a specialist insurance policy in place which would operate in the event of any participant suffering harm as a result of their involvement in the research (Newline Underwriting Management Ltd, at Lloyd's of London). NHS indemnity operates in respect of the clinical treatment that is provided.

19.3 Contractual Arrangements

Appropriate contractual arrangements will be put in place with all third parties.

20 PUBLICATION POLICY

The findings will be published in high impact, peer-reviewed journals with open access and with full acknowledgement of the support provided by NIHR/NETSCC/EME. A full and complete account of the research will also be published in the relevant NIHR programme journal.

The primary report will be attributed to the PAX-D Investigators and collaborators. The names of all investigators who enter a participant into the randomised phase will be listed at the end of the primary publication.

Wider media dissemination will be planned and coordinated through the BRC Communications Team with patient and public involvement provided by the Oxford Health BRC Patients and Research Group. This will include systematic reviews, guidelines and evidence syntheses. Health economic analyses and results will be reported to field conferences and journals, oral communications, posters, symposia and keynote lectures at national and international psychiatric conferences and smaller specialist meetings at research institutions.

21 DATA SHARING

De-identified individual clinical trial participant-level data will be made available for sharing in ethically approved individual patient data synthesis and meta-analyses on receipt of appropriate application and subject to approval by data sharing committee (to be convened during trial).

22 DEVELOPMENT OF A NEW PRODUCT/ PROCESS OR THE GENERATION OF INTELLECTUAL PROPERTY

Ownership of IP generated by employees of the University vests in the University. The protection and exploitation of any new IP is managed by the University's technology transfer office, Oxford University Innovations.

23 ARCHIVING

Archiving will follow the completion of the study and publication of results. All essential documents will be retained for a minimum of 5 years after the date of resolution of the last data query. At this point, the requirement to continue to archive these documents will be reviewed in line with applicable data protection guidelines.

24 REFERENCES

Aiken, C. B. (2007). Pramipexole in psychiatry: a systematic review of the literature. J Clin Psychiatry, 68(8), 1230–6.

Al-Janabi H, Flynn T, Coast J. Development of a self-report measure of capability wellbeing for adults: the ICECAP-A. Quality of Life Research, 2012, 21: 167-176.

Bharmal M, Payne K, Atkinson MJ, Desrosiers M-P, Morisky DE, Gemmen E. Validation of an abbreviated Treatment Satisfaction Questionnaire for Medication (TSQM-9) among patients on antihypertensive medications. Health Qual Life Outcomes. 2009; 7: 36.

Browning, Michael, Timothy E. Behrens, Gerhard Jocham, Jill X. O'Reilly, and Sonia J. Bishop. 2015. Anxious Individuals Have Difficulty Learning the Causal Statistics of Aversive Environments'. Nature Neuroscience 18 (4): 590–96.

Burdick, K. E., Braga, R. J., Gopin, C. B., & Malhotra, A. K. (2014). Dopaminergic influences on emotional decision making in euthymic bipolar patients. Neuropsychopharmacology: Official Publication of the American College of Neuropsychopharmacology, 39(2), 274–282.

Chan TY. (2000) Improvements in the packaging of drugs and chemicals may reduce the likelihood of severe intentional poisonings in adults. Hum Exp Toxicol. 2000 Jul;19(7):387-91

Chisolm D, Knapp M, Knudsen H, et al., Client Socio-Demographic and Service Receipt Inventory - European Version : development of an instrument for international research, The British Journal of Psychiatry, 2000, 177, s28-s33.

Cools, R. (2008). Role of dopamine in the motivational and cognitive control of behavior. The Neuroscientist: A Review Journal Bringing Neurobiology, Neurology and Psychiatry, 14(4), 381–395.

Cools, R., Altamirano, L., & D'Esposito, M. (2006). Reversal learning in Parkinson's disease depends on medication status and outcome valence. Neuropsychologia, 44(10), 1663–1673.

Corrigan, M. H., Denahan, A. Q., Wright, C. E., Ragual, R. J., & Evans, D. L. (2000). Comparison of pramipexole, fluoxetine, and placebo in patients with major depression. Depression and Anxiety, 11(2), 58–65.

Curtis, L. & Burns, A. (2017) Unit Costs of Health and Social Care 2017, Personal Social Services Research Unit, University of Kent, Canterbury.

Cusin, C., Iovieno, N., Iosifescu, D. V., Nierenberg, A. A., Fava, M., Rush, A. J., & Perlis, R. H. (2013). A randomized, double-blind, placebo-controlled trial of pramipexole augmentation in treatment resistant major depressive disorder. The Journal of Clinical Psychiatry, 74(7), e636-641.

Drummond ME, Sculpher MJ, Claxton K, Stoddart GL, Torrance GW, editors. Methods for the economic evaluation of health care programmes. Oxford: Oxford University Press; 2015.

Efron B, Tibshirani RJ. An introduction to the bootstrap. New York: Chapman Hall; 1993.

EuroQol Group. EuroQol--a new facility for the measurement of health-related quality of life. Health Policy 1990;16(3):199-208.

Fawcett, J., Rush, A. J., Vukelich, J., Diaz, S. H., Dunklee, L., Romo, P.,Escalona, R. (2016). Clinical Experience With High-Dosage Pramipexole in Patients With Treatment-Resistant Depressive Episodes in Unipolar and Bipolar Depression. The American Journal of Psychiatry, 173(2), 107-111.

Fenwick E, Byford S. A guide to cost-effectiveness acceptability curves. British Journal of Psychiatry 2005; 187: 106-8.

Flynn T. N., Huynh E., Peters T. J., Al-Janabi H., Clemens S., Moody A. and Coast J. Scoring the Icecap-a Capability Instrument. Estimation of a UK General Population Tariff, Health Econ., 2015; 24; 258–269.

Fostick, L., Silberman, A., Beckman, M., Spivak, B., & Amital, D. (2010). The economic impact of depression: resistance or severity? European Neuropsychopharmacology: The Journal of the European College of Neuropsychopharmacology, 20(10), 671–675.

Franco-Chaves JA, Mateus CF, Luckenbaugh DA, Martinez PE, Mallinger AG, Zarate CA (2013) Combining a dopamine agonist and selective serotonin reuptake inhibitor for the treatment of depression: A double blind, randomized pilot study. J Affect Disord. 149:319-325.

Fritz, M. S., & MacKinnon, D. P. (2007). Required Sample Size to Detect the Mediated Effect. Psychological Science, 18(3), 233–239.

Geddes et al. Lithium plus valproate combination therapy versus monotherapy for relapse prevention in bipolar I disorder (BALANCE): an randomised open-lable trial. Lancet 2009, 375: 385-95.

Geddes, John R., Alexandra Gardiner, Jennifer Rendell, Merryn Voysey, Elizabeth Tunbridge,Christopher Hinds, Ly-Mee Yu, et al. 2016. 'Comparative Evaluation of Quetiapine plus Lamotrigine Combination versus Quetiapine Monotherapy (and Folic Acid versus Placebo) in Bipolar Depression (CEQUEL): A 2 × 2 Factorial Randomised Trial'. The Lancet. Psychiatry 3 (1): 31–39.

Goldberg JF, Burdick KE, Endick CJ. Preliminary randomized, double-blind, placebo-controlled trial of pramipexole added to mood stabilizers for treatment-resistant bipolar depression. Am J Psychiatry 2004: 161(3):564-6.

Hawton K, Ware C, Mistry H, Hewitt J, Kingsbury S, Roberts D, Weitzel H. (1996) Paracetamol self-poisoning. Characteristics, prevention and harm reduction. Br J Psychiatry. Jan;168(1):43-8.

Herdman M, Gudex C, Lloyd A, Janssen MF, Kind P, Parkin D, Bonsel G, Badia X. Development and preliminary testing of the new five-level version of EQ-5D (EQ-5D-5L). Quality of Life Research. 2011 20(10):1727-36.

Joint Formulary Committee. (2018). British national formulary 75. London: BMJ Publishing and the Royal Pharmaceutical Society.

Little, R.J. and D.B. Rubin. Statistical Analysis with Missing Data. 2nd ed. Wiley Series in Probability and Statistics. Hoboken, NJ: Wiley, 2002.

Mitchell PM, et al. Assessing the validity of the ICECAP-A capability measure for adults with depression. BMC Psychiatry 2017;17(1):46.

Morriss, R., Garland, A., Nixon, N., Guo, B., James, M., Kaylor-Hughes, C.NIHR CLAHRC Specialist Mood Disorder Study Group. (2016). Efficacy and cost-effectiveness of a specialist depression service versus usual specialist mental health care to manage persistent depression: a randomised controlled trial. The Lancet. Psychiatry, 3(9), 821–831. https://doi.org/10.1016/S2215-0366(16)30143-2

Mundt, J., Marks, I., Shear, M., & Greist, J. (2002). The Work and Social Adjustment Scale: A simple measure of impairment in functioning. British Journal of Psychiatry, 180(5), 461-464.

Newton, T. F., Haile, C. N., Mahoney, J. J., Shah, R., Verrico, C. D., De La Garza, R., & Kosten, T. R. (2015). Dopamine D3 receptor-preferring agonist enhances the subjective effects of cocaine in humans. Psychiatry Research, 230(1), 44–49. https://doi.org/10.1016/j.psychres.2015.07.073

NICE 2009a Depression in adults: recognition and management: Clinical guideline [CG90] Published date: October 2009 Last updated: April 2016

NICE (National Institute for Health and Care Excellence). Managing long-term sickness absence and incapacity for work. 2009b, London: National Institute for Health and Clinical Excellence.

NICE (National Institute for Health and Care Excellence). Guide to the methods of technology appraisal. 2013, London: National Institute for Health and Care Excellence.

Patel A, McCrone P, Leese M, Amaddeo F, Tansella M, Kilian R, Angermeyer M, Kikkert M, Schene A Knapp M. Cost-effectiveness of adherence therapy versus health education for people with schizophrenia: randomised controlled trial in four European countries. Cost Effectiveness and Resource Allocation 2013; 11: 12.

Pulcu, E., & Browning, M. (2017)a. Affective bias as a rational response to the statistics of rewards and punishments. ELife, 6. https://doi.org/10.7554/eLife.27879

Pulcu, Erdem, and Michael Browning. 2017b. ‘Using Computational Psychiatry to Rule Out the Hidden Causes of Depression’. JAMA Psychiatry 74 (8): 777–78.

Richard I. H, Kurlan R. (1997). A survey of antidepressant drug use in Parkinson's disease. Parkinson Study Group. Neurology, 49(4):1168-70.

Rush, A. J., Trivedi, M. H., Wisniewski, S. R., Nierenberg, A. A., Stewart, J. W., Warden, D., Fava, M. (2006). Acute and longer-term outcomes in depressed outpatients requiring one or several treatment steps: a STAR*D report. The American Journal of Psychiatry, 163(11), 1905–1917.

Schulz, K. F. Subverting randomisation in controlled trials. JAMA 274, 1456-1458. 1995.

Schultz, W., Dayan, P., & Montague, P. R. (1997). A neural substrate of prediction and reward. Science, 275(5306), 1593–9.

Shelton, R. C., & Tomarken, A. J. (2001). Can recovery from depression be achieved? Psychiatric Services (Washington, D.C.), 52(11), 1469–1478.

Simon J, et al. Operationalising the capability approach for outcome measurement in mental health research. Soc Sci Med 2013;98:187-96.

Simon J, Vergunst F, Jenkinson C, Anand P, Gray A, Rugkåsa J, Yeeles K, Burns T. (2014). The OxCAP-MH: A novel, multi-dimensional, patient-reported capabilities instrument for mental health research and policy. The Journal of Mental Health Policy and Economics. 17. S21.

Simon J, Mayer S (2016) HEQ - Health Economics Questionnaire, Version v5.0 (08-09-2016), Department of Health Economics, Center for Public Health, Medical University of Vienna, Vienna.

Simon J, Mayer, S (2021) HEQ COVID-19, Version 24-02-2021, Department of Health Economics, Center for Public Health, Medical University of Vienna, Vienna.

Snaith RP, Hamilton M, Morley S, Humayan A, Hargreaves D, Trigwell P. A scale for the assessment of hedonic tone the Snaith-Hamilton Pleasure Scale.Br J Psychiatry. 1995 Jul;167(1):99-103.

Spitzer et al. (2006) A Brief Measure for Assessing Generalized Anxiety Disorder: The GAD-7. Arch. Intern. Med 166: 1092-1097.

Steptoe A, Breeze E, Banks J, Nazroo J. Cohort profile: the English longitudinal study of ageing. Int J Epidemiol. 2013;42:1640–8.

Stinnett A, Mullahy J. Net health benefits: a new framework for the analysis of uncertainty in cost-effectiveness analysis. Medical decision making: an international journal of the Society for Medical Decision Making 1998; 18: s68-s80.

Tarricone, R. Cost-of-illness analysis: What room in health economics? Health Policy 2006; 77(1): 51-63.

Tundo A, de Filippis R, and De Crescenzo F (2019) Pramipexole in the treatment of unipolar and bipolar depression. A systematic review and meta-analysis. Acta Psychiatr Scand. 2019 May 20 doi: 10.1111/acps.13055.

Weintraub D, Mamikonyan E, Papay K, Shea JA, Xie SX, Siderowf A. Questionnaire for Impulsive-Compulsive Disorders in Parkinson's Disease-Rating Scale. Mov Disord. 2012 Feb;27(2):242-7

White IR, Royston P, Wood AM. Multiple imputation using chained equations: Issues and guidance for practice. Stat Med. 2011; 30(4): 377-99.

Zarate CA, Jr., Payne JL, Singh J, Quiroz JA, Luckenbaugh DA, Denicoff KD, et al. Pramipexole for bipolar II depression: a placebo-controlled proof of concept study. Biol Psychiatry 2004: 56(1):54-60.

Zhou, X., Ravindran, A. V., Qin, B., Del Giovane, C., Li, Q., Bauer, M., … Xie, P. (2015). Comparative efficacy, acceptability, and tolerability of augmentation agents in treatment-resistant depression: systematic review and network meta-analysis. The Journal of Clinical Psychiatry, 76(4), 487.

Date and version No: Version 3, 24/08/2021

25 APPENDIX A: SCHEDULE OF PROCEDURES – CLINIC VISITS

	**Screening c. Day -14**	**Randomisation Week 0**	**Week 2 +/- 3 days**	**Week 6 +/- 4 days**	**Week 12 +/- 4 days**	**Week 48 +/- 4 days**
Informed consent	✓					
Demographics	✓					
Clinical diagnosis	✓					
Medical history	✓					
Blood pressure, pulse	✓	✓	✓	✓	✓	✓
Urine Pregnancy Test (WOCBP only)	✓	✓				✓
Height	✓					
Weight	✓				✓	✓
Venepuncture	Serum Creatinine	Research samples				
Eligibility assessment	✓	✓				
Randomisation		✓				
Decision Making Task	✓ (Trial run)	✓	✓		✓	
QIDS-C	✓	✓	✓	✓	✓	✓
AE check	✓	✓	✓	✓	✓	✓[1]
Concomitant medication	✓	✓	✓	✓	✓	✓
Post-trial treatment arranged						✓

Notes:

[1] Safety follow-up is required for all participants for at least two weeks after pramipexole treatment has been stopped (see section 12.5).

Au-Yeung SK, *et al. Evid Based Ment Health* 2022; 25:77–83. doi: 10.1136/ebmental-2021-300282

Date and version No: Version 3, 24/08/2021

26 APPENDIX B: SCHEDULE OF PROCEDURES – RA CONTACT AND SELF REPORTING

RA contact between trial visits			
	**Initiation**	**Weeks 0 – 12**	**Weeks 13 - 48**
Checks for AEs suicidality, medication changes	Screening	✓ Weekly	✓ 4-weekly
QUIP-RS	Screening	✓ Weekly	✓ 4-weekly
TSQM	Week 1 (after commencing trial medication)	Weeks 1, 6, 12, 24 and 48	

Self-reporting via True Colours			
	**Initiation of scale**	**Weeks 0 – 12**	**Weeks 13 - 48**
QIDS-SR_{16}	Screening	✓ Weekly	✓ 4-weekly
ALTMAN	Screening	✓ Weekly	✓ 4-weekly
GAD-7	Screening	✓ Weekly	✓ 4-weekly
SHAPS	Screening	✓ 2- weekly	✓ 4-weekly
UCLA Loneliness	Randomisation	✓ Weeks 6, 12	✓ Week 48
ELSA Social Isolation	Randomisation	✓ Weeks 6, 12	✓ Week 48
WSAS	Randomisation	✓ 4-weekly	✓ Weeks 24, 36 and 48
EQ-5D-5L	Randomisation	✓ Week 12	✓ Weeks 24, 36 and 48
ICECAP-A	Randomisation	✓ Week 12	✓ Weeks 24, 36 and 48
OxCAP-MH	Randomisation	✓ Week 12	✓ Weeks 24, 36 and 48
HEQ	Randomisation	✓ Week 12	✓ Weeks 24, 36 and 48

Au-Yeung SK, *et al. Evid Based Ment Health* 2022; 25:77–83. doi: 10.1136/ebmental-2021-300282

27 APPENDIX C: DECISION-MAKING TASK

The decision-making task (Figure A4) has been adapted from a structurally similar learning task previously reported in the literature (Pessiglone et al. 2006). The task involves 3 runs of 60 trials each (180 trials in total). On each trial of the task participants are presented with two abstract shapes (letters selected from the Agathodaimon font) and choose the shape which they believe will result in the best outcome. Two shapes may result in either winning 20 or 0 points (with one shape leading to a win of 20 points on 70% of trials and the other shape on 30% of trials) while a separate pair of shapes may result in losing 20 or 0 points (with one shape leading to a loss of 20 points on 70% of trials and the other shape leading to a loss on 30% of trials). Participants must learn from the outcome of previous trials what they think the best shape to choose is.

In total, the task takes approximately 15 minutes to complete. It is administered online, using the True Colours system. Choice data from the task is analysed both by calculating accuracy scores and by fitting a behavioural model which is described below.

Behavioural Model Used in Analysis of the Learning Task: The primary measure of interest in the learning task is the sensitivity for wins and for losses, although it also allows separation of this parameter from the learning rate. A simple behavioural model, based on that employed in related tasks is used to first estimate learning rate. This model first estimates the separate value for each shape. When that shape is chosen its value is updated using:

$$\boldsymbol{q_{(s,i+1)} = q_{(s,i)} + \alpha * (outcome_{(s,i)} - q_{(s,i)})}$$

In these equations $q_{(s,i)}$, which is initialised at 5, is the estimated value for shape s on trial $outcome_{(s,i)}$ is a variable coding for whether the outcome of the trial (1 for a win, 0 for no change and -1 for a loss) and α is a free parameter, the learning rate (separate learning rates will be used for wins and losses). The value of the unchosen option will not change. These estimated values are then transformed into a single choice probability using a soft max function:

$$\boldsymbol{PchoiceA_{(i)} = \frac{exp^{\beta(q_{(s,i)})}}{exp^{\beta(q_{(s,i)})} + exp^{\beta(q_{(\sim s,i)})}}}$$

Where $PchoiceA_{(i)}$ is the probability of choosing shape “A” on trial i, and β is the outcome sensitivities. As for learning rate, separate outcome sensitivities will be estimated for win and loss trials. The four free-parameters of this model (learning rates and sensitivities for wins and losses) are estimated separately for each participant by calculating the full joint posterior probability of the parameters, given participants’ choices, and then deriving the expected value of each parameter from their marginalised probability distributions (Browning 2015).

Au-Yeung SK, *et al. Evid Based Ment Health* 2022; 25:77–83. doi: 10.1136/ebmental-2021-300282

Date and version No: Version 3, 24/08/2021

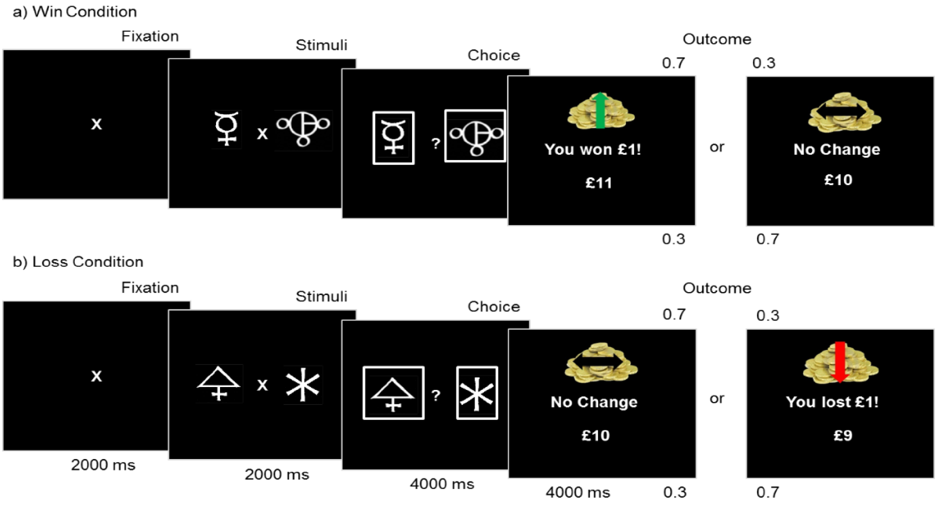

Figure A4. Task structure Timeline of one trial from the decision making task. Participants are presented with two shapes and have to choose one. Each trial is either a "win" trial or a "loss" trial. On win trials one shape will result in winning 20 points 70% of the time and the 0 points for the other 30% (the other shape has the opposite probabilities). On loss trials one shape will result in losing 20 points 70% of the time and losing 0 points for the other 30% (with the other shape having the opposite probability). Using trial and error participants learn which are the best shapes to choose to maximise overall winnings.

Au-Yeung SK, *et al. Evid Based Ment Health* 2022; 25:77–83. doi: 10.1136/ebmental-2021-300282

28 APPENDIX D: QUESTIONNAIRES/RATING SCALES

Questionnaires/rating scales (including measures of depression, mania, pleasure, anxiety and impulse control) will be administered through a combination of participant self-reports via True Colours (see section 10.4), semi-structured interviews (see section 10.8.2), and completion by a psychiatrist during a clinic visit.

Quick Inventory of Depressive Symptomatology (QIDS-SR$_{16}$)

This is a 16-item self-report questionnaire that covers 9 key symptoms of depression (Rush, 2003). The scale is designed to assess both severity of depression and change in depressive symptoms over time. Participants are instructed to score each item according to the description that best describes how they have been over the past 7 days. Each of the symptoms is scored on a 4-point scale (0 – 3) giving a maximum score (after combining of some items) of 27. In addition to inclusion of the total score in analyses, Item 12 will be used to detect suicidal thoughts.

The QIDS-SR$_{16}$ will be completed online using True Colours (see section 10.4).

Quick Inventory of Depressive Symptomatology (QIDS-C)

As for the QIDS-SR above, rated by a clinician during study visits.

ALTMAN Self-Report Scale for mania (ALTMAN)

The ASRM is a 5-item self-report questionnaire that assesses severity of and change in manic symptoms (Altman 1997). Participants are instructed to score each item according to the description that best describes how they have been over the past 7 days. Each of the symptoms is scored on a 5-point scale (0 – 4) giving a maximum score of 20.

The ALTMAN will be completed online using True Colours (see section 10.4).

General Anxiety Disorder Scale (GAD-7)

The GAD-7 is 7-item self-report questionnaire that can be used to screen for anxiety disorder and to measure severity (Spitzer, 2006). Assessment is derived from the total score across all 7 items.

The GAD-7 will be completed online using True Colours (see section 10.4).

Snaith-Hamilton Pleasure Scale (SHAPS)

The SHAPS is a 14-item scale that measures anhedonia, the inability to experience pleasure (Snaith et al, 1995). The items cover the domains of: social interaction, food and drink, sensory experience, and interest/pastimes. Each item has four possible responses: strongly disagree, disagree, agree, or strongly

agree. Either of the "disagree" responses score one point, and either of the "agree" responses score 0 points. The final score ranges from 0 to 14, with higher scores indicating higher levels of anhedonia.

The SHAPS will be completed online using True Colours (see section 10.4).

UCLA Loneliness Scale

The UCLA 3-item Loneliness Scale was created in 2004, and is considerably shorter and easier to administer than the original 20-item instrument. This scale comprises 3 questions that measure three dimensions of loneliness: relational connectedness, social connectedness and self-perceived isolation. The scale generally uses three response categories: Hardly ever (scoring 3) / some of the time (scoring 2) / often (scoring 1). The scores for each individual question can be added together to give you a possible range of scores from 3 to 9.

The UCLA Loneliness Scale will be completed online using True Colours (see section 10.4).

ELSA Social Isolation Measure

The ELSA Social Isolation Measure is derived from the English Longitudinal Study of Ageing (ELSA), a nationally representative panel study of people aged 50 years or older living in England (Steptoe et al, 2013). It is a widely used measure of social isolation, often employed with some modifications. In the PAX-D trial, the whole scale will be collected for the baseline measure, then for weeks 6, 12 and 48 the first 3 response options will be collected (3+ times per week, 1-2 per week, 1-2 per month) with a change of the 4th response to 'not in the past month'.

The ELSA Social Isolation Measure will be completed online using True Colours (see section 10.4).

Treatment Satisfaction Questionnaire for Medication (TSQM)

The 9-item version of the TSQM (Bharmal et al, 2009) assesses the overall level of satisfaction or dissatisfaction with medication patients are taking. The domains covered are, adverse events, symptom relief, convenience, effectiveness, impact on daily life, and tolerability/acceptability. Each item is rated on a 7-point scale from 'Extremely Satisfied' to 'Extremely Dissatisfied'.

The TSQM will be completed by the RA during semi-structured interviews (see section 10.8.2).

Questionnaire for Impulsive-Compulsive Disorders in Parkinson's Disease–Rating Scale (QUIP-RS)

The QUIP-RS (Weintraub et al, 2012) has four primary questions (pertaining to commonly reported thoughts, urges/desires, and behaviours associated with impulse control disorders), each applied to the four impulse control disorders (compulsive gambling, buying, eating, and sexual behaviour) and 3 related disorders (medication use, spending and hobbyism). It uses a 5-point Likert scale (score 0–4 for each

question) to gauge the frequency of behaviours, and instructs patients to answer questions based on behaviours that occurred in the preceding 4 weeks (or any 4-week period in a designated time frame).

The QUP-RS will be completed by the RA during semi-structured interviews (see section 10.8.2).

Work and Social Adjustment Scale (WSAS)

The WSAS (Mundt et al, 2002) is a simple measure of impairment in functioning, and assesses the impact of a respondent's mental health difficulties on their ability to function in terms of five dimensions (work, home management, social leisure, private leisure, and personal or family relationships). Severity is measured on an eight-point Likert scale (ranging from 'not at all' to 'very severely'. The total WSAS score is derived by adding the scores across all the items.

The WSAS will be completed online using True Colours (see section 10.4).

EQ-5D-5L

The EQ-5D-5L is a standardised measure of health status developed by the EuroQol group, and provides a simple generic measure of health for clinical and economic appraisal (Herdman et al, 2011). The scale has five dimensions (mobility, self-care, usual activities, pain/discomfort, and anxiety/depression) and five levels for each dimension (no problems, slight problems, moderate problems, severe problems, extreme problems. A visual scale records the respondent's self-rated health with endpoints labelled 'the best health you can imagine' and 'the worst health you can imagine'.

The EQ-5D-5L will be completed online using True Colours (see section 10.4).

ICEpop CAPability measure for Adults (ICECAP-A)

The ICECAP-A is a measure of capability for the general adult (18+) population for use in economic evaluation (Al-Janabi et al, 2012). Unlike most profile measures used in economic evaluations, the ICECAP-A focuses on wellbeing defined in a broader sense, rather than health. The measure covers attributes of wellbeing that were found to be important to adults in the UK. This capabilities scale has 5 dimensions (attachment, stability, achievement, enjoyment, and autonomy) and assesses broader well-being.

The ICECAP-A will be completed online using True Colours (see section 10.4).

Oxford CAPabilities questionnaire-Mental Health (OxCAP-MH)

This capabilities scale is a mental health specific well-being scale with 16 items (Simon et al, 2013). The items cover dimensions of wellbeing (overall health, social and recreational activities, loss of sleep due to worry, friendship and support, having suitable accommodation, feeling safe, likelihood of discrimination and assault, freedom of personal and artistic expression, appreciation of nature, self-determination, and access to activities or employment). Each response is scored on a 5-point Likert scale.

The OxCAP-MH will be completed online using True Colours (see section 10.4).

Health Economics Questionnaire (HEQ)

The Health Economics Questionnaire has specifically been developed for mental health economic evaluations and is an adapted version of the widely-used and validated CSSRI instrument for collection of resource use data in mental health (Simon & Mayer, 2016). The questionnaire measures health and social care resource use, medication, absenteeism from work and presenteeism as well as socio-demographic background information. The HEQ was subsequently updated to allow for resource-use measurement in times of pandemics (Simon and Mayer, 2021). The HEQ COVID-19 now both reflects the changed service provision landscape and having direct information on any COVID-19 related service use.

The HEQ COVID-19 will be completed online using True Colours (see section 10.4).

29 APPENDIX E: AMENDMENT HISTORY

Amendment No.	Protocol Version No.	Date issued	Author(s) of changes	Details of Changes made
Amendment 4	3.0	24/08/2021	James Griffiths, Michael Browning	Description of Lindus Health services added to Recruitment section.
Amendment 3	2.0	15/07/2021	James Griffiths, Michael Browning	Changes to questionnaire schedules for QIDS-SR16, QUIP-RS, GAD-7, ALTMAN, WSAS and TSQM; reflecting initial experience and to correct inconsistency in previous version of protocol. Details of blood sample collection updated. Information added on monitoring of allocation concealment and blinding.<br>Updated details for Trial Statistician and TSC Chair. Clarified that medically qualified staff may be delegated to prescribe by the PI and do not need to be consultant psychiatrists.
n/a	1.2	29/10/2019	James Griffiths, Michael Browning	Qualification of contraceptive advice (abstinence). Appendix A amended to reflect duration of safety follow-up. Submitted to MHRA in response to grounds for non-acceptance.
n/a	1.1	25/09/2019	James Griffiths, Michael Browning	Addition of UCLA 3-point Loneliness Scale and ESLA Social Isolation Scale. Additional detail on questionnaire completion.
n/a	1.0	05/09/2019	n/a	First version. Approved by Sponsor but not submitted for REC or MHRA approval.

Details of all protocol amendments will be included here whenever a new version of the protocol is produced.
